# Artificial neural network approach for prediction of AuNPs biosynthesis by *Streptomyces flavolimosus*, characterization, antitumor potency in-vitro and in-vivo against Ehrlich ascites carcinoma 

**DOI:** 10.1038/s41598-023-39177-4

**Published:** 2023-08-04

**Authors:** Noura El-Ahmady El-Naggar, Nashwa H. Rabei, Mohamed F. Elmansy, Omar T. Elmessiry, Mostafa K. El-Sherbeny, Mohanad E. El-Saidy, Mohamed T. Sarhan, Manar G. Helal

**Affiliations:** 1 Department of Bioprocess Development, Genetic Engineering and Biotechnology Research Institute, City of Scientific Research and Technological Applications (SRTA-City), New Borg El-Arab City, 21934 Alexandria Egypt; 2 Biotechnology and Its Application Program, Department of Botany, Faculty of Science, Mansoura University, Mansoura, 35516 Egypt; 3 Department of Pharmacology and Toxicology, Faculty of Pharmacy, Mansoura University, Mansoura, 35516 Egypt

**Keywords:** Nanoparticles, Applied microbiology

## Abstract

Gold nanoparticles (AuNPs) have emerged as promising and versatile nanoparticles for cancer therapy and are widely used in drug and gene delivery, biomedical imaging, diagnosis, and biosensors. The current study describes a biological-based strategy for AuNPs biosynthesis using the cell-free supernatant of *Streptomyces flavolimosus*. The biosynthesized AuNPs have an absorption peak at 530–535 nm. The TEM images indicate that AuNPs were spherical and ranged in size from 4 to 20 nm. The surface capping molecules of AuNPs are negatively charged, having a Zeta potential of − 10.9 mV. FTIR analysis revealed that the AuNPs surface composition contains a variety of functional groups as –OH, C–H, N–, C=O, NH_3_^+^, amine hydrochloride, amide group of proteins, C–C and C–N. The bioprocess variables affecting AuNPs biosynthesis were optimized by using the central composite design (CCD) in order to maximize the AuNPs biosynthesis. The maximum yield of AuNPs (866.29 µg AuNPs/mL) was obtained using temperature (35 °C), incubation period (4 days), HAuCl_4_ concentration (1000 µg/mL) and initial pH level 6. Comparison was made between the fitness of CCD versus Artificial neural network (ANN) approach based on their prediction and the corresponding experimental results. AuNPs biosynthesis values predicted by ANN exhibit a more reasonable agreement with the experimental result. The anticancer activities of AuNPs were assessed under both in vitro and in vivo conditions. The results revealed a significant inhibitory effect on the proliferation of the MCF-7 and Hela carcinoma cell lines treated with AuNPs with IC_50_ value of 13.4 ± 0.44 μg/mL and 13.8 ± 0.45 μg/mL for MCF-7 and Hela cells; respectively. Further, AuNPs showed potential inhibitory effect against tumor growth in tumor-bearing mice models. AuNPs significantly reduced the tumor volume, tumor weight, and decreased number of viable tumor cells in EAC bearing mice.

## Introduction

Nanoparticles have several potential applications that can help improve human life and its environment^1^. Nanoparticles have unique physicochemical characteristics compared to their solid bulk materials because of their greater surface area to volume ratio, shape, size, electronic properties, and high reaction activity^2^. Owing to their characteristics, nanoparticles have several applications in diverse fields^3^.

Bulk gold (Au) is regarded as a metal that is non-reactive or inert to several chemical reactions. However, gold nanoparticles (AuNPs) have a number of unique physicochemical characteristics. These characteristics include the electronic characteristics, the localized surface Plasmon resonance, the novel unique characteristics with the quantum size effects, and the change in individual localized energy levels^3^. Numerous and well-documented applications of gold nanoparticles in the biomedical sciences, including drug and gene delivery^4^, treatments for cancer^5^, medicine and surgery^6^, bio-imaging^7^, biosensors^8^, the development of specific scaffolds, transplacental treatment, and amyloid-like fibrillogenesis inhibitors^9^. AuNPs are promising candidates for molecular diagnostics^10^. In addition, gold nanoparticles have wide biomedical applications, including rheumatoid arthritis treatment, photothermal therapy, antiviral, and antibacterial agents^11^. AuNPs could be potentially effective as therapeutic agents for treating diabetes and associated microvascular complications based on their potential as anti-hyperglycemic, antioxidant, anti-inflammatory, antiglycation, diabetic wound healing, anti-fibrotic, and antiangiogenic agents^12^.

Traditionally, gold nanoparticles have been synthesized using chemical and physical techniques. However, their use has a number of disadvantages. The principal disadvantages of chemical techniques include the use of very hazardous chemicals, environmental pollution, and carcinogenic solvents, which restrict their use in the clinical field. Physical approaches require expensive equipment and high energy consumption. In addition, the low stability of AuNPs, the difficulty in controlling crystal formation, and particle aggregation are disadvantages of the previous strategies^13^. Green synthesis of AuNPs is an effective strategy due to crucial aspects such as low-cost production, use of non-toxic reducing agents without additional stabilizers, production of size-controlled nanoparticles, use of renewable resources, and low energy consumption^13,14^. Currently, the green synthesis of nanoparticles using biological resources that are readily available in nature, such as plant extracts or microorganisms like actinomycetes, fungi, bacteria, and algae as potential nano-factories has been reported^15,16^. Microbial cells secrete numerous enzymes that cause the reduction of metal ions^17^. Microorganisms or their protein extracts are capable of producing nanoparticles in a renewable manner without using any external chemicals, as they act as bio-reducers, capping/stabilizing agents, or both throughout the nanoparticles production process^18,19^. Actinomycetes serve as excellent sources for the biosynthesis of nanoparticles that exhibit desirable surface and size properties because they produce a diverse array of secondary metabolites^20^.

The present study aims to prepare gold nanoparticles using *Streptomyces flavolimosus* as a biological method, to optimize the bioprocess variables affecting AuNPs biosynthesis using the central composite design (CCD) in order to maximize the AuNPs biosynthesis, to employ an artificial neural network (ANN) approach for analyzing CCD, validating and predicting AuNPs biosynthesis. A comparison was made between the fitness of CCD versus ANN models and the evaluation of the biosynthesized AuNPs for their potential for cancer treatment under both in vitro and in vivo conditions.

This is the first report on artificial intelligence-based optimization of AuNPs using the cell-free supernatant of *Streptomyces flavolimosus* as a novel source for the actinomycete-mediated synthesis of gold nanoparticles.

## Materials and methods

### Microorganisms and cultural conditions

The strain of *Streptomyces flavolimosus* used in the current study was kindly donated by the first author. *Streptomyces flavolimosus* was grown and maintained on Petri plates that contained medium of starch-nitrate agar containing the following ingredients(g/L): Starch 20; CaCO_3_ 3; K_2_HPO_4_ 1; KNO_3_ 2; MgSO_4_.7H_2_O 0.5; NaCl 0.5; FeSO_4_.7H_2_O 0.01; distilled water up to one liter and agar was 20 g/L. The Petri dishes were incubated at 30 °C for 7 days. The spore suspension of *Streptomyces flavolimosus* was preserved in glycerol (20%, v/v) at − 20 °C.

### Inoculum preparation

*Streptomyces flavolimosus* culture was grown on starch nitrate agar plates for seven days at 30 °C. After that, three discs with a diameter of 9 mm were taken from a well grown culture and inoculated into 250 mL Erlenmeyer flasks that contained 50 mL of sterile medium consisting of the following ingredients (g/L): Soluble starch 20; yeast extract 0.3; KNO_3_ 1; MgSO_4_⋅7 H_2_O 0.5; NaCl 0.5; K_2_HPO_4_ 0.5; FeSO_4_⋅7H_2_O 0.01 and distilled water up to 1 L. pH 7–7.5. The inoculated flasks were incubated for 48 h under aerobic conditions in an incubator shaker at 30 °C, 150 rpm. The obtained culture served as inoculum for subsequent experiments.

### Extracellular synthesis of AuNPs

In order to biosynthesize AuNPs, *Streptomyces flavolimosus* was grown in an Erlenmeyer flask containing 50 mL of sterile medium of the following ingredients (g/L): Soluble starch 20; yeast extract 0.3; KNO_3_ 1; MgSO_4_⋅7 H_2_O 0.5; NaCl 0.5; K_2_HPO_4_ 0.5; FeSO_4_⋅7H_2_O 0.01 and distilled water up to 1 L. pH 7–7.5. The flasks were incubated for 48 h under aerobic conditions in an incubator shaker at 150 rpm at the temperature of 30–37 °C for 5 days. Biosynthesis of AuNPs has been performed using the cell-free supernatant of *Streptomyces flavolimosus*. In this study, 1000 µg/mL stock solution of HAuCl_4_.7H_2_O was prepared by dissolving 0.2041 g of the salt in 100 mL of distilled water. After adding varying amounts of HAuCl_4_ solution to cell-free supernatant at a ratio of 1:2 volume to volume, the mixture was placed in the dark in an incubator shaker at 37 °C for 24–72 h. As a control, the cell-free supernatant was held without addition of HAuCl_4_ solution. After the incubation time, the color of the mixture changed to red or dark purple, indicating the reduction of gold chloride and the biosynthesis of gold nanoparticles in the solution. To verify the reduction of gold chloride and the biosynthesis of gold nanoparticles, the UV–visIble absorbance of the solution sample was scanned in the range of 450 and 650 nm in a UV–Visible spectrophotometer.

### Characterization of AuNPs

#### UV–Visible spectrum

In order to determine the wavelength of maximum absorbance, biosynthesized AuNPs were analyzed by UV–Vis spectroscopy.

The biosynthesized AuNPs were characterized by scanning them with an Optizen Pop-UV/Vis spectrophotometer over the wavelength range of 450 to 650 nm.

#### TEM analysis of AuNPs sample

AuNPs size, shape and morphology were investigated using TEM (JEOL-JEM-2100 Plus, Ltd., Japan). EDX (Energy Dispersive X-ray) spectroscopy analysis was used to determine the elemental composition of a sample. Mapping analysis was performed using TEM to demonstrates the composition and distribution of AuNPs sample. Also, selected area electron diffraction (SAED) was investigated using TEM.

#### Zeta-potential of the synthesized AuNPs

There is no reliable method for determining the surface charge of tiny particles in liquid. The zeta potential is a highly significant metric for determining the behavior of colloids or nanoparticles when they are suspended. Its value is highly correlated with particle surface shape and suspension stability. The ζ-potential of AuNPs was quantified with a Malvern 3000 Zetasizer Nano ZS, UK.

#### Fourier transform infrared (FTIR) spectroscopy analysis

FTIR spectroscopy analysis was utilized in order to determine the biomolecules responsible for reducing, capping, and stabilizing agents of AuNPs and to investigate the surface properties of AuNPs. The FTIR spectra of the cell-free supernatant and AuNPs were determined in a range between 4500 and 500 cm^−1^ with a resolution of 1 cm^−1^.

#### XRD pattern

The crystalline nature of the AuNPs and structural characteristics were analyzed using X-ray diffraction (XRD) measurement using advanced X-ray diffractometer (Bruker D2 Phaser 2nd Gen) equipped with a CuKα radiation, λ = 1.5406 Å source (applied voltage 10 kV, current 30 mA). XRD spectrum of AuNPs was obtained at 2*θ* between 10 and 70 and a scanning rate of 2°/min.

#### Optimization of AuNPs by central composite design (CCD)

Using CCD, the optimum levels of 4 independent variables and their effects on AuNPs biosynthesis were determined. The four variables vary on three different levels. The tested independent variables were temperature (25–45 °C), incubation period (2–6 days), different concentration of HAuCl_4_ (200–1000 µg/mL) and pH levels (4–8). Thirty experimental trials in which 6 runs were conducted at the central levels. The relations among the process independent variables and AuNPs biosynthesis, µg/mL (the response) were identified by applying the polynomial equation of the second degree.

1 $$Y = \beta_{0} + \sum\limits_{i} {\beta_{i} X_{i} } + \sum\limits_{ii} {\beta_{ii} X_{i}^{2} } + \sum\limits_{ij} {\beta_{ij} X_{i} X_{j} } .$$

In which Y is the predicted (AuNPs biosynthesis, µg/mL), (X_i_) is the coded values of the independent factors, (β_0_) represents the regression coefficients, (β_ij_) the interaction coefficients, the linear coefficient (β_i_), and quadratic coefficients (β_ii_).

#### Artificial neural network (ANN) analysis

CCD of the four process factors and AuNPs biosynthesis by using the cell-free supernatant of *Streptomyces flavolimosus* were employed to ANN analysis. The ANN analysis was performed using JMP pro 14 Software. The ANN analysis is composed of three layers, a layer of input comprising the four independent components, hidden layer and a single-neuron output layer (biosynthesis of AuNPs, g/mL). Comparison was made between the fitness of CCD and ANN models based on their prediction and the corresponding experimental results. Evaluation of efficacy and the model selection was determined based on R^2^ values, SSE, MAD and RMSE.

### Statistical analysis

Both the experimental design and statistical analyses were performed with Design Expert 12 for Windows (https://www.statease.com/software/design-expert/). In order to generate three-dimensional surface plots, the STATISTICA 8 software was utilized (https://www.statsoft.de/de/software/statistica). JMP pro 14 Software was used to conduct the artificial neural network (ANN) analysis (https://www.jmp.com/en_in/home.html).

### Assessment of in vitro cytotoxicity and anticancer activities

The anticancer activities of AuNPs synthesized by using the cell-free supernatant of *Streptomyces flavolimosus* were measured in vitro on both breast cancer (MCF-7) and cervical epithelioid carcinoma (Hela) cell lines using standard 3-(4, 5 dimethythiazol-2-yl)-2, 5-diphenyl tetrazolium bromide (MTT) assay^21^. The source of these cancer cell lines is VACSERA in Cairo, Egypt. The carcinoma cells were treated with various concentrations of AuNPs (100, 25, 6.25, 1.56, and 0.39 µg/mL), and Doxo was chosen as a comparative anticancer agent. The absorbance was measured using an ELISA microplate reader (Bioline Eliza Reader). The background absorbance of multiwell plates was measured at 690 nm and was subtracted from the 450 nm measurement. The half maximal inhibitory concentration (IC_50_) is defined as the drug concentration that can inhibit 50% of cell growth inhibition. It was calculated using nonlinear regression fitting models and the equation for the Boltzman sigmoidal concentration–response curve by using GraphPad Prism Version 9 Software.

### Assessment of in vivo antitumor activities against Ehrlich ascites carcinoma (EAC)

#### Ethics statement

All experimental protocols were approved by Research Ethics Committee, “Animal Care and Use Committee”, Mansoura University, Egypt. All the experiments were performed in accordance with the relevant guidelines and regulations. The authors complied with the ARRIVE guidelines.

#### Chemicals

The Egyptian International Medical Center for Pharmaceuticals (EIMC) supplied 2 mg/mL Doxo. The chemicals in this experiment were all of analytical grade.

#### Animals

Adult Swiss female albino mice (n = 32), weighing 20–25 g, were obtained from VACSERA, Giza, Egypt. The mice were fed with a standardized chow diet with free access to water ad libitum, housed on a 12/12-h light/dark cycle.

#### EAC cell line

The EAC cell line was procured from Cairo University’s National Cancer Institute (NCI), Cairo, Egypt.

### Experimental design

To initiate the EAC, 200 µL of cells (5 × 10^5^ viable cells/mL) implanted subcutaneously at the right thigh of mice^22^. Five days following tumor implantation (day zero), solid tumor can be observed. There were four distinct groups of animals (8 mice/each) as follows:

Group 1; EAC control group: This group consisted of mice that were given normal saline by oral administration for a period of 21 days. Group 2; EAC/Doxo group: This group consisted of mice that were injected intraperitoneally (I.P.) with Doxo (2 mg/kg/day) for 21 days^23^. Group 3; EAC/AuNPs group: mice were treated orally with AuNPs (5 mg/kg/day) for 21 days. AuNPs was dissolved in normal saline immediately prior to use and its dose was selected based on trial experiment. Group 4; EAC/Doxo/AuNPs group: mice were I.P. injected with Doxo (2 mg/kg/day) and were treated orally with AuNPs (5 mg/kg/day) for 21 days.

Using a digital caliper, tumor volumes were measured in two dimensions every 5 days. These measurements were starting on day zero (the day before the therapy began), day 5, day 10, day 15, and day 20. At the end of the experiment, (on day 21 of treatment), mice were sacrificed by cervical dislocation under anesthesia using thiopental sodium (40 mg/kg). The tumor masses were detached, weighed, and stored in buffered formalin solution for subsequent histopathological examination.

### The determination of the tumor’s volume, weight, and the percentage of growth inhibition

The anticancer activity was evaluated by determining the tumor’s volume as well as its weight. The volume of the tumor was determined using the formula A × B^2^ × 0.5. A represents the diameter of the largest diameter of the tumor and B represents its perpendicular. At the end of the experiments, the weight of each individual tumor mass was recorded. The following formula was employed to determine the tumor growth inhibition rate^24^:

$${\text{Mean tumor weight of EAC control group }} - {\text{ Mean tumor weight of treated group}}/{\text{Mean tumor weight of EAC control group}} \times {1}00.$$

### Histopathological analysis of the tissue

The fixed tumors were then embedded in paraffin and sectioned to a thickness of 5 μm. After being stained with Hematoxylin and Eosin, the sections were examined using a light microscope.

### Statistical analysis

The data were analyzed using a one-way analysis of variance (ANOVA), and then by Tukey’s post-hoc test. The data were expressed as the mean accompanied by the standard error of the mean. The *P*-values that were lower than 0.05 were regarded as being statistically significant.

## Results and discussion

**Figure 1:**
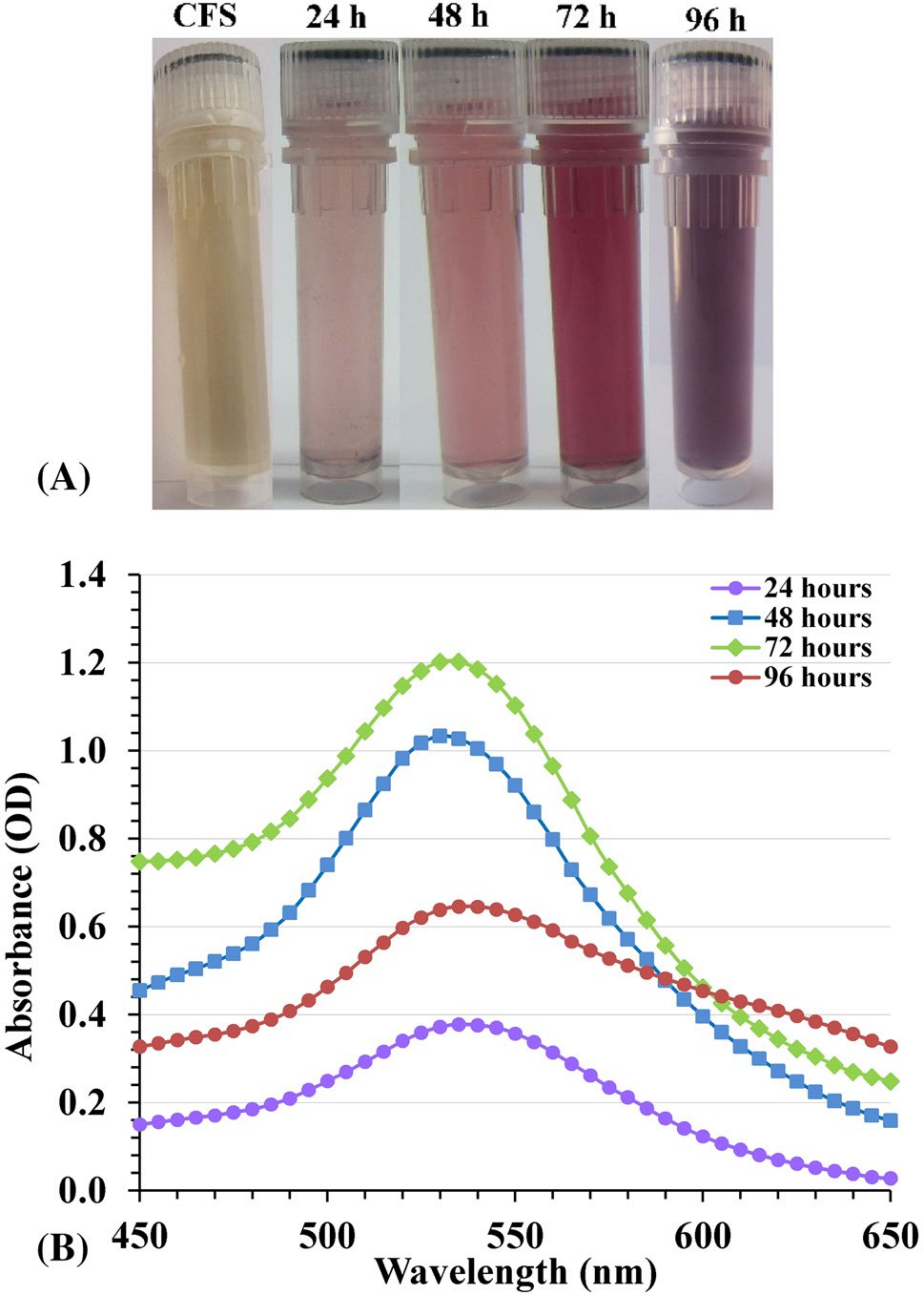
(**A**) Vials of CFS and AuNPs solution at different time intervals; (**B**) UV/visible spectrum of the biosynthesized AuNPs (a single SPR band at 530–535 nm). CFS: cell-free supernatant.

There is a growing demand to develop eco-friendly, non-toxic, cost-effective and reliable routes of gold nanoparticles synthesis. The biosynthesis of AuNPs has been accomplished using the cell-free supernatant of *Streptomyces flavolimosus*. The cell-free supernatant was treated with aqueous solution of HAuCl_4_. The reaction mixture was incubated in an incubator shaker in the dark condition to avoid photolytic reaction at 37 °C for 24–72 h. After the incubation time, the reduction of HAuCl_4_ to AuNPs was monitored visually and the change in the color of the reaction mixture from yellow, violet to red or dark purple was observed, indicating the reduction of gold chloride and formation of AuNPs (Fig. 1A). In contrast, no colour change was observed in a control mixture when the cell-free supernatant was incubated under the same conditions without the addition of aqueous HAuCl_4_ solution.

The mechanism of the biogenic process is not fully understood. The biosynthesis process of AuNPs takes place in two steps: at the first, the gold ions (Gold (III) chloride, traditionally called auric chloride, HAuCl_4_, chloroauric acid, Au^3+^) are transformed to AuNPs (Au^0^) in presence of reducing agents, and followed by the coating of synthesized AuNPs. During the green synthesis processes, a wide variety of organic as well as biological molecules (enzymes, phenols, sugars, etc.) present in biological samples can participate both in the gold ions reduction and in the sufficient capping and stabilization of the prepared nanoparticles^13,25^. It is believed that enzymatic synthesis is one of the most efficient possible methods for producing AuNPs. The enzyme nitrate reductase was found to catalyze and play an essential role in the process of gold ions reduction^13^.

### UV–Visible spectral analysis

AuNPs biosynthesis was explored using UV–Visible spectroscopy in the spectral region 450–650 nm, with the progression of the period. Characterization of AuNPs begins with a colour change based on the surface Plasmon resonance principle (SPR). SPR is the optical property that is unique to metallic nanoparticles. The metal surface carry free electrons in the conduction band and the nuclei are positively charged. SPR is caused by the oscillations of electrons in the conduction band close to the nanoparticles’ surface. The specific oscillations modes of electrons limited by the particles size and shape. Consequently, metallic nanoparticles exhibit distinctive optical absorption spectra in the ultraviolet–visible range. When the size of the particles increases, a color change takes place; in the case of gold, this change goes from a deep red to a purple color. The SPR is responsible for the variations in color observed during AuNPs biosynthesis and UV–Visible spectroscopy is used to determine the absorbance of the various color changes. The optical properties of AuNPs–from light pink to dark red-are controlled by their morphology, which includes their size, shape, the degree to which they aggregate and structural characteristics of these nanoparticles^26^.

To identify the effect of the cell-free supernatant on AuNPs biosynthesis, the absorbance spectrum of the cell-free supernatant was performed (Supplementary Fig. S1). SPR spectrum of AuNPs synthesized using *Streptomyces flavolimosus* cell-free supernatant showed the presence of a distinct absorption peak with maximum absorbance wavelength located at 530–535 nm (Fig. 1B). It is identical to the SPR spectra of AuNPs. The results indicate that complete reduction of the HAuCl_4_ ions occurs after nearly 3 days of the reaction incubation time. Ahmad et al.^27^ reported that the SPR band of AuNPs solution’s staying at 530 nm during the duration of the reaction period proves that there is no sign of aggregation and that the particles are dispersed in the aqueous solution. Complete reduction of the HAuCl_4_ ions takes approximately 120 h, demonstrating that this is a very slow process^27^. Doan et al.^28^ observed that the presence of a single absorption peak exhibiting maximum absorbance at around 535 nm is attributable to the AuNPs SPR band, and the concentration of gold ions have a significant impact on the AuNPs biosynthesis. The increase in UV–Vis absorbance was caused by the increase in gold ion concentration. Kalabegishvili et al.^29^ reported that, the synthesis of AuNPs was caused by the addition of *Streptomyces glaucus* 71MD biomass to chloroauric acid and the maximum absorption peak indicating the biosynthesis of gold nanoparticles was observed at 530 nm. On the other hand, Khadivi Derakhshan et al.^30^ stated that UV visible absorption spectrum of AuNPs produced with *Streptomyces griseus* cells exhibited a prominent and broad peak at 540 nm.

After three days of the reaction time, a decrease in the absorbance was noticed in the intensity of the SPR band at 96 h. Also, the absorbance spectrum become broadened at 96 h indicating the possibility of AuNPs agglomeration. Sobczak-Kupiec et al*.*^31^ reported that shifting as well as broadening of the surface plasmon band (SPB) of gold nanoparticles, reveals the formation of gold nanoparticles with nearly constant size distribution. The absorbance shift indicates the interlayer aggregation of gold nanoparticles. On long time reaction, the initially formed particle serves as a nucleation site and as a result layer by deposition of gold takes place resulting in the increase in size^31^. On the other hand, Gupta et al*.*^32^ observed that with increasing of time, the AuNPs agglomerate and fuse to develop into larger crystals, and thus large-sized AuNPs are produced.

### Transmission electron microscopy (TEM) analysis

**Figure 2:**
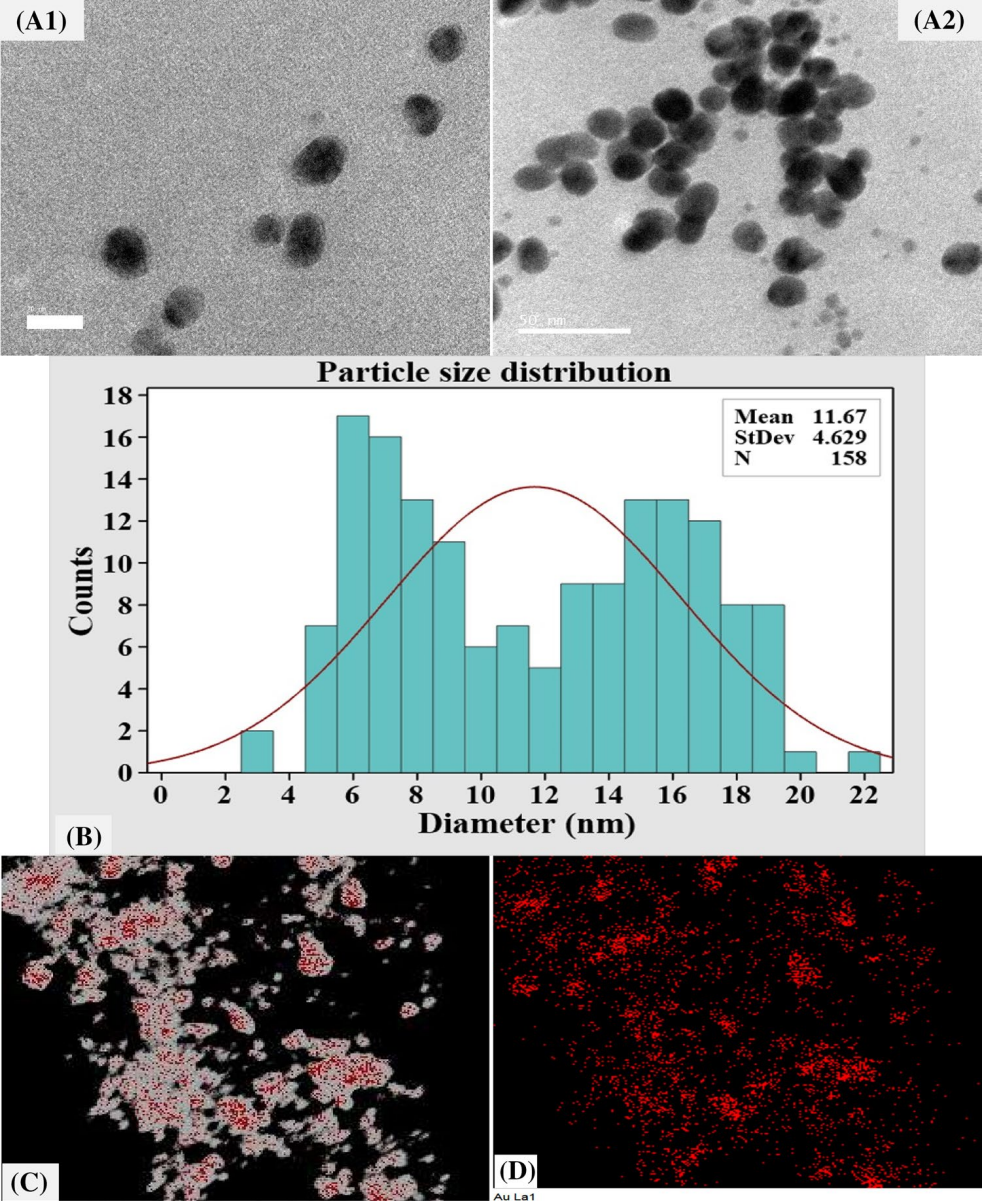
The biosynthesized AuNPs, as detected by the micrographs of TEM (**A1**, **A2**), particle size distribution (**B**) and mapping analysis (**C**, **D**). The scale bar for (**A1**, **A2**) is 20 and 50 nm, respectively.

The morphology, microstructures, and size of the biosynthesized AuNPs were determined by TEM analysis. TEM is an effective tool for investigating nanoparticles and is widely recognized for examining morphological characteristics, such as shape, size, and surface area^33^. Figure 2A demonstrates that TEM investigation revealed the formation of well-dispersed spherical AuNPs with sizes ranging from 4 to 20 nm. Whereas Fig. 2B displays a plot of the histogram of particle size distribution that was calculated based on an analysis of 158 particles with mean size distribution of 11.67 nm.

Protocols for the biological synthesis of gold nanoparticles provide a clean and eco-friendly method for the production of nanoparticles with a broad range of sizes, shapes, physico-chemical and biological properties^34^. Huang et al.^35^ reported that characteristic size-distribution and TEM images of the AuNPs have disclosed spherical shaped AuNPs with a broad spectrum of particle size with the size ranging from 5 to 50 nm. Additionally, Cai et al.^36^ reported that TEM image for AuNPs biosynthesized by *Magnetospirillum gryphiswaldense* MSR-1 show spherical nanoparticles, with an irregular size distribution in the range of 5–40 nm. Rajeshkumar^37^ reported that TEM investigation for AuNPs biosynthesized by marine bacteria *Enterococcus* sp. demonstrated the formation of spherical shaped AuNPs with size ranging from 6 to 13 nm.

Mapping analysis of AuNPs was carried out to investigate the distribution and the composition of biosynthesized AuNPs. TEM elemental mapping analysis results illustrate the whole distribution of AuNPs and its components (Au) (Fig. 2C).

**Figure 3:**
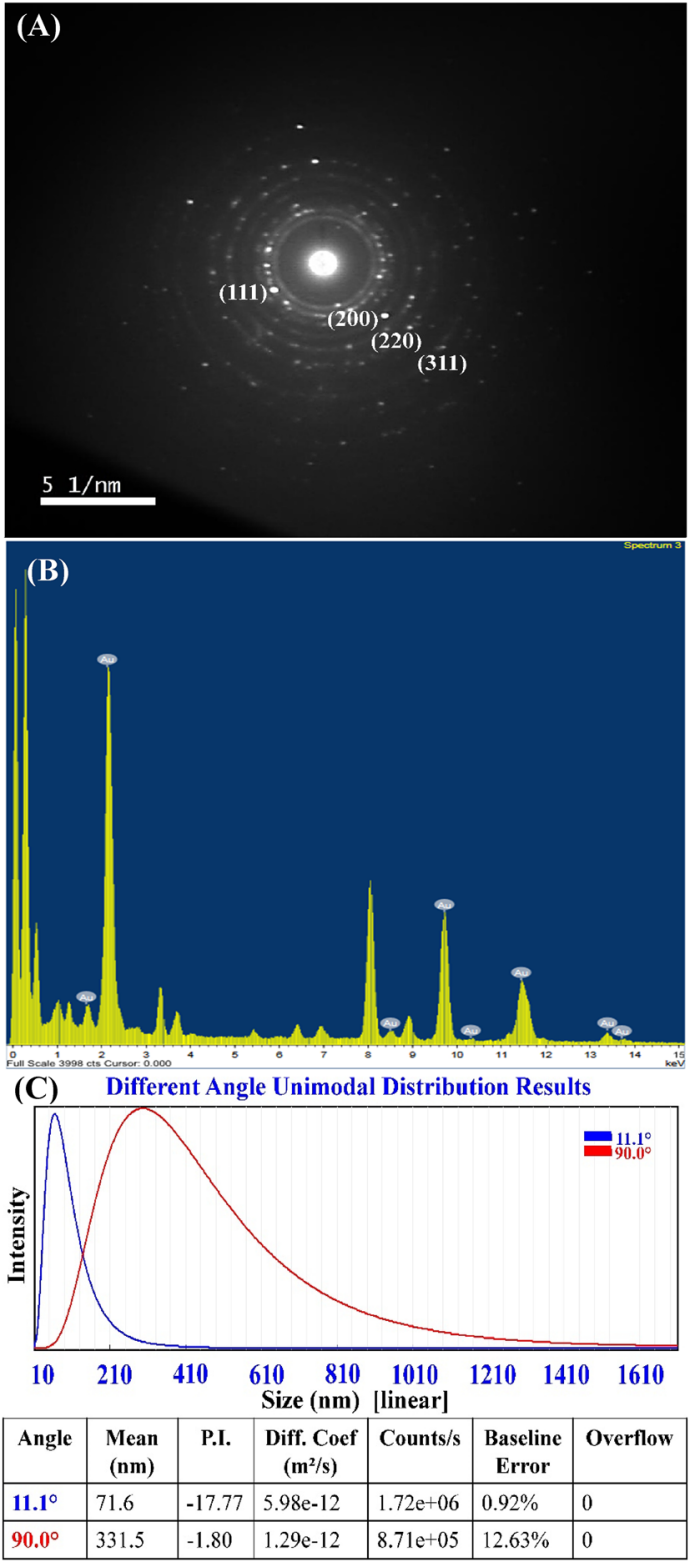
Selected area electron diffraction (SAED) (**A**) and EDX analysis (**B**) and particle size analyzer measurement (**C**) of the biosynthesized AuNPs.

To confirm AuNPs formation and its crystalline structure, SAED (selected area electron diffraction pattern) analysis was conducted (Fig. 3A). SAED patterns obtained by TEM were used to evaluate the crystallinity of the AuNPs^38^. AuNPs crystalline structure was demonstrated by the bright circular well-separated diffraction rings related to the following four lattice planes: (111), (200), (220), and (311), which indicated the fcc (face-centered cubic) structure of the gold^38^.

Figure 3B shows standard EDX spectrum of the biosynthesized AuNPs. EDX by TEM is an analytical method used to determine the elemental composition of a sample^33^. The existence of gold in the produced AuNPs was confirmed by EDX analysis^39^.

### Particle size analysis

Particle Size Analyzer (PSA) was used in this investigation to assess the particle size distribution of an AuNPs suspension. The particle size distribution was determined at ambient temperature over a range of wavelengths from 1 to 760 nm. As shown in Fig. 3C, a particle size analyzer measurement of a sample of AuNPs revealed a narrow and sharp peak at 71.6 nm diameter at *θ* = 11.1° and 331.5 nm at *θ* = 90°. The sizes that were measured by the particle size analyzer can only be used as a relative value, and cannot be compared to those determined by electron microscopy^40^. Electron microscopy is able to determine the shape and surface structure of the particles and their geometric dimensions by measuring the width of individual particles from the image^41,42^. Imaging was favored because of its high-resolution particle’s visualization and negligible impact of artifacts on size determination^42^.

### Zeta potential (ζ) measurement

**Figure 4:**
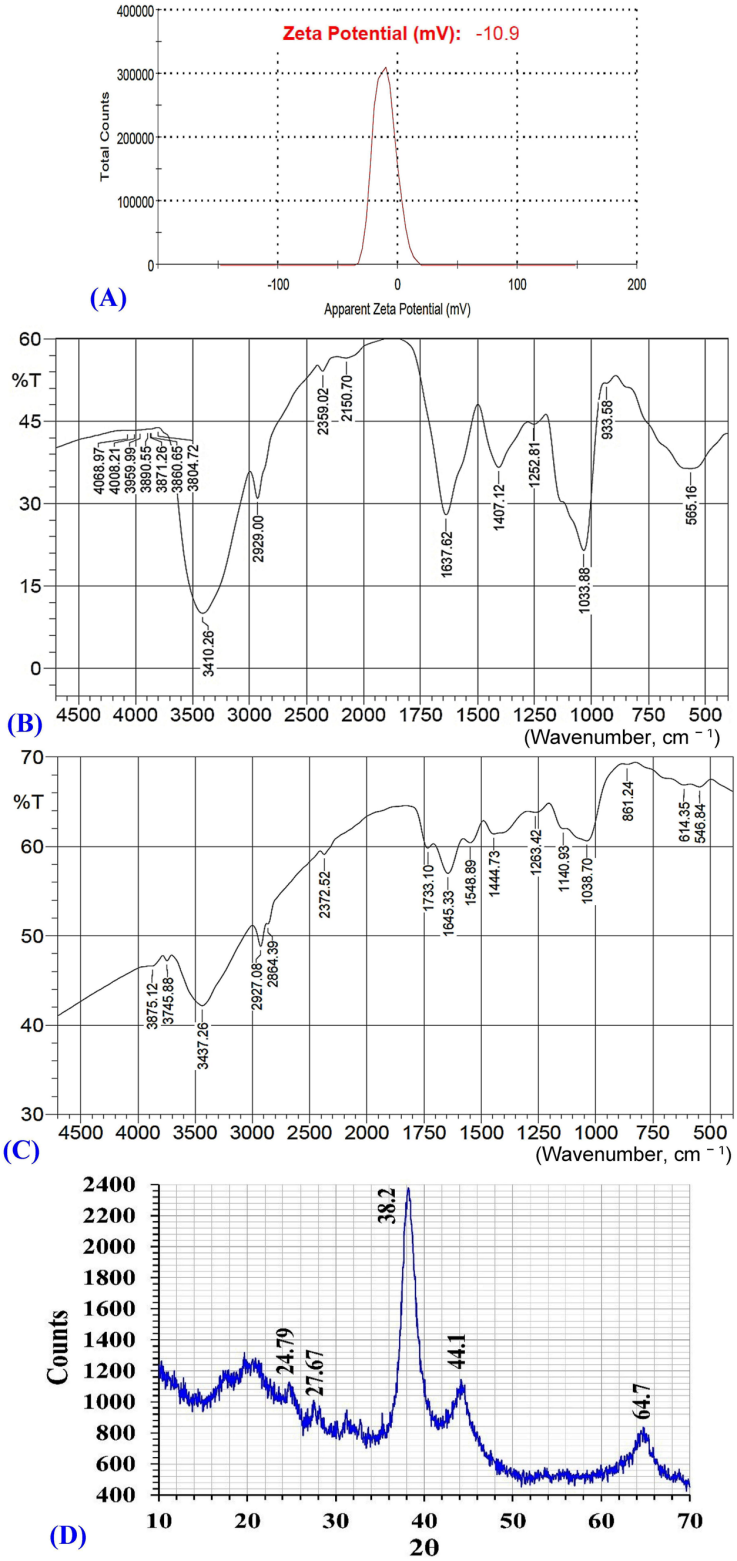
Analyses of the biosynthesized AuNPs with (**A**) zeta potential, (**B**) FTIR of AuNPs, (**C**) FTIR of cell-free supernatant and (**D**) XRD.

The net charge of nanoparticles is a key characteristic that influences their stability and dispersion^33^. Therefore, the zeta potential (ζ) values were used to determine the stability and the surface charge of the biosynthesized AuNPs. Zeta potential is measured depending on the velocity and direction of particles in a well-defined electric field^43^. Bhambure et al*.*^44^ reported that zeta potential defines the particle’s repelling forces, which prevent aggregation and is responsible for stabilization of the synthesized AuNPs in the solution. Figure 4A demonstrates that the biosynthesized AuNPs showed zeta potential value of − 10.9 mV. The negative zeta potential value indicates that AuNPs are surrounded by negatively charged organic molecules, which reduces the repulsion between the AuNPs, prevents their aggregation, and subsequently increases their stability^45^.

The zeta potential value was found to be − 9.8 mV^46^. The synthesized AuNPs showed negative zeta potential value of − 2.26 mV^45^. Two different types of AuNPs synthesized from *Cannabis sativa* showed zeta potential values of − 12.3 mV and − 20.6 mV^46^. The high values of zeta potential mean that AuNPs are highly stable due to the presence of high surface charge that prevent aggregation and ensure redispersion due to repulsive electric forces. At values of zeta potential, aggregation may form^47^. As a general rule, zeta potential value ≥ 30 mV is considered good and excellent stability^47^. Values of zeta potential ≥  ± 30 mV indicates monodisperse formulations without aggregates^48^, while zeta potential value ~  ± 20 mV is prone to have only short-term stability, and zeta potential value < 5 mV tends to aggregate rapidly^47^.

Since zeta potential is influenced by temperature, solvent viscosity, pH, ionic strength, and surface characteristics, even minor parameter variations can significantly change its absolute value^49^. Moreover, changing the concentration of gold salt used for the synthesis reaction, pH, and temperature can also provide control over the size and geometry of AuNPs^50^.

The stable nanoparticles are stabilized in presence of stabilizing agents. Various studies have reported that proteins, terpenoids, phenolic compounds, and nicotinamide adenine dinucleotide might act as capping and stabilizing agents during AuNPs biosynthesis^50^. According to the findings of Muthuvel et al*.*^43^, the capping molecules that are found on the surface of AuNPs are predominantly made up of groups that have a negative charge. The various functional groups such as carboxylic, polyphenol, alcohol, alkene, primary amine, and surfactant presence in proteins may control the shape and size of nanoparticles with the change of salt precursors, pH, and temperature^25^. The stabilization of NPs done by using various capping agents can be divided into three stabilization processes, including steric stabilization, electrostatic stabilization, and unification of steric and electrostatic stabilization^25,51^.

### Fourier transformed infrared analysis (FTIR)

Green synthesis of AuNPs was achieved using the cell-free supernatant of *Streptomyces flavolimosus* as a biocatalyst for reduction of HAuCl_4_ and capping/stabilizing of the AuNPs. The FTIR analysis was carried out to determine the biomolecules responsible for reducing, capping, and stabilizing agents of AuNPs. FTIR analysis was performed in order to identify the functional groups situated on the AuNPs’ surface^52^ or identifying the probable biomolecules responsible for the reduction of Au^+^ ions by the cell-free supernatant^53^.

FTIR spectrum of the bio-synthesized AuNPs was analyzed and compared with the FTIR spectrum of the cell-free supernatant of *Streptomyces flavolimosus* (Fig. 4B,C). The FTIR spectrum of the cell-free supernatant of *Streptomyces flavolimosus* (Fig. 4B) reveals absorption peaks at 3745, 3437, 2927, 2372, 1733, 1645, 1548, 1444, 1263, 1140, 1038, 861, 614 and 546 cm^−1^. Significant shifts of peaks in the spectrum of AuNPs from peaks in the spectrum of the cell-free supernatant of *Streptomyces flavolimosus* (Fig. 4C) indicate a significant role of various function groups in the cell-free supernatant of *Streptomyces flavolimosus* in the reduction, capping, and stabilizing AuNPs. The FTIR spectrum of AuNPs (Fig. 4C) reveals distinct absorption peaks at 3410, 2929, 2359, 2150, 1637, 1407, 1252, 1033, 933 and 565 cm^−1^.

Hydroxyl (OH) stretching peak at wavelength 3437 cm^−1^ found in the spectrum of cell-free supernatant of *Streptomyces flavolimosus* shifted to 3410 cm^−1^ which is attributed to –OH stretching^54^ on the formation of AuNPs. Moreover, the absorption corresponding to C–H stretching vibration was observed at 2927 cm^−1^ found in the spectrum of cell-free supernatant of *Streptomyces flavolimosus* shifted to 2929 cm^−1^ which is associated with the C–H stretching of alkanes^55^ on the formation of AuNPs. The C=O or the N–H stretching vibrations were thought to be responsible for the peak that was seen at approximately 2359 cm^−1^^56^. The band located at 2150 cm^−1^ could be assigned to C=O^57^, it could also be attributed to amine hydrochloride stretching vibration and the presence of NH_3_^+^^58^.

The peaks at 1645 cm^−1^ found in the spectrum of cell-free supernatant of *Streptomyces flavolimosus* corresponds to amide I due to C=O stretching of α-helix proteins^59^ shifted on the formation of AuNPs to the absorption peak situated at 1637 cm^−1^ assigned to the amide group I, and it is caused by the stretching vibrations of carbonyl that are present in proteins amide linkage^60^. On the other hand, the presence of peak at 1548 cm^−1^ in the FTIR spectrum of cell-free supernatant of *Streptomyces flavolimosus* is due to the N–H bending vibrations of the protein amide II group. The peak at 1548 cm^−1^ disappeared in the FTIR spectrum after AuNPs biosynthesis, reveals that this groups is involved in the AuNPs biosynthesis by the culture filtrate of *Streptomyces flavolimosus*.

The C–C stretching of aromatic molecules is represented by the bands at 1407 cm^−1^^55^ and C–O stretching vibrations^61^. Additionally, the peak of the C–N stretching vibrations seen in the spectral range of 1250 to 1000 cm^−1^^62^. The band observed at 1252 cm^−1^ is assigned to the C–C stretching vibrations^63^. The absorption peaks at 1038 cm^−1^ found in the spectrum of cell-free supernatant of *Streptomyces flavolimosus* could be due to the stretching vibration of the C–O groups in the monosaccharides shifted on the formation of AuNPs to the absorption peaks at 1033 cm^−1^ corresponds to the C–O stretching vibrations that are present in various biocomponents^64^. The peak at 933 cm^−1^, is indicative of hydrogen bridging those results from the generation of carboxylic acid dimers^65^.

### X-ray diffraction pattern (XRD)

XRD pattern of AuNPs biosynthesized by using the cell-free supernatant of *Streptomyces flavolimosus* was performed (Fig. 4D). The peaks of AuNPs were found at 2*θ* = 24.79°, 27.67°, 38.2°, 44.1°, 64.7° (Fig. 4D), in the range of 20°–70°. The typical peaks of AuNPs were located at 2*θ* = 38.1°, 44.3°, 64.6°, 77.6°, 81.7°, 98.2°, 111.0° and 115.2°^66^. Doan et al.^28^ stated that the maximum peak of diffraction for AuNPs occurring at 2*θ* angle of approximately 38.2° indicated that the crystals have a favoured growth orientation in the plane (111) and the result obtained clearly proves that the AuNPs formed were crystalline in nature^67^.

### Statistical optimization of AuNPs biosynthesis using the cell-free supernatant of *Streptomyces flavolimosus* strain using central composite design (CCD)

Many factors, including incubation time, temperature, pH, and HAuCl_4_ concentration, can have an effect on the size, distribution, and quantity of gold nanoparticles that are synthesized. In the current study, the effects of four factors on AuNPs biosynthesis (as a response) were explored. These four variables were the HAuCl_4_ concentration, the initial pH level, temperature, and the incubation period.

**Figure 5:**
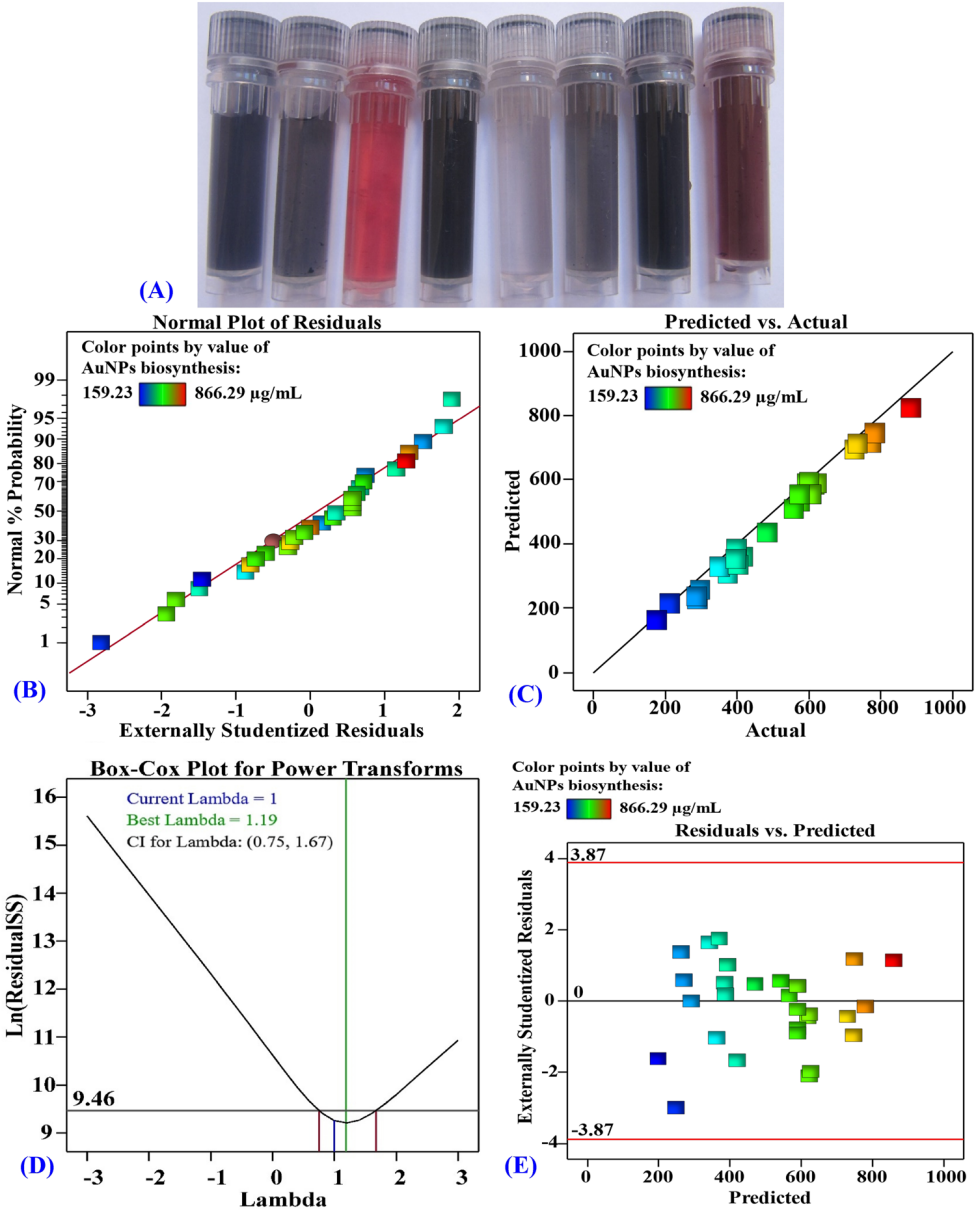
(**A**) Photographs of the AuNPs solutions show color changes as affected by the levels of different independent variables during the optimization process. (**B**) NPP of internally studentized residuals, (**C**) plot of predicted versus actual, (**D**) Box–Cox plot for power transforms and (**E**) plot of the residuals versus predicted values of the biosynthesized AuNPs using the cell-free supernatant of *Streptomyces flavolimosus*.

The bioprocess variables affecting the AuNPs biosynthesis were optimized by using the CCD in order to maximize the AuNPs biosynthesis and to investigate the quadratic, interaction, and individual effects of the process variables on the biosynthesis of AuNPs by using the cell-free supernatant of *Streptomyces flavolimosus*. In order to determine the optimum values for the variables of interest, a CCD consisting of thirty separate trials was applied. Table 1 illustrates the design matrix containing the four investigated variables, their actual and coded levels, as well as the experimental and predicted biosynthesized AuNPs values and their residual values. The results that were obtained from the CCD experiments for the biosynthesized AuNPs using the cell-free supernatant of *Streptomyces flavolimosus* reveal that there are significant color changes reflected by the biosynthesized AuNPs as affected by the levels of different independent variables during the optimization process (Fig. 5A).

**Table 1:** CCD and ANN of process factors with coded and actual levels, experimental and predicted values of AuNPs biosynthesis using the cell-free supernatant of *Streptomyces flavolimosus*.

Std	Run	Type	X_1_	X_2_	X_3_	X_4_	AuNPs biosynthesis (µg/mL)				
							Actual	CCD		ANN	
								Predicted	Residuals	Predicted	Residuals
2	1	Fact	1	− 1	− 1	− 1	356.17	328.06	28.11	360.58	4.41
22	2	Axial	0	0	2	0	866.29	845.4	20.88	870.36	4.07
15	3	Fact	− 1	1	1	1	604.77	609.52	− 4.75	605.58	0.81
27	4	Center	0	0	0	0	559.57	575.71	− 16.14	576.26	16.69
3	5	Fact	− 1	1	− 1	− 1	272.23	247.95	24.28	268.04	− 4.19
26	6	Center	0	0	0	0	588.63	575.71	12.91	576.26	− 12.37
13	7	Fact	− 1	− 1	1	1	709.33	715.06	− 5.73	709.66	0.33
16	8	Fact	1	1	1	1	717.77	732.56	− 14.78	716.99	− 0.78
17	9	Axial	− 2	0	0	0	540.2	528.51	11.69	540.75	0.55
8	10	Fact	1	1	1	− 1	556.34	551.85	4.5	556.66	0.32
6	11	Fact	1	− 1	1	− 1	756.36	734.86	21.49	755.98	− 0.38
1	12	Fact	− 1	− 1	− 1	− 1	382	371.1	10.9	381.04	− 0.96
9	13	Fact	− 1	− 1	− 1	1	398.72	379.85	18.87	397.25	− 1.47
12	14	Fact	1	1	− 1	1	385.23	355.71	29.51	384.85	− 0.38
4	15	Fact	1	1	− 1	− 1	278.68	276.84	1.85	256.93	− 21.75
30	16	Center	0	0	0	0	556.34	575.71	− 19.37	576.26	19.92
24	17	Axial	0	0	0	2	378.77	373.48	5.29	380.08	1.31
18	18	Axial	2	0	0	0	577.35	608.52	− 31.17	583.70	6.35
21	19	Axial	0	0	− 2	0	194.83	235.19	− 40.37	195.48	0.65
10	20	Fact	1	− 1	− 1	1	269.59	257.52	12.06	269.06	− 0.53
25	21	Center	0	0	0	0	588.63	575.71	12.91	576.26	− 12.37
5	22	Fact	− 1	− 1	1	− 1	598.31	604.46	− 6.15	598.54	0.23
11	23	Fact	− 1	1	− 1	1	380.74	406.11	− 25.37	381.75	1.01
19	24	Axial	0	− 2	0	0	583.02	612.6	− 29.58	585.24	2.22
14	25	Fact	1	− 1	1	1	765.25	766.16	− 0.9172	761.20	− 4.05
28	26	Center	0	0	0	0	572.48	575.71	− 3.23	576.26	3.78
7	27	Fact	− 1	1	1	− 1	333.57	349.51	− 15.94	335.31	1.74
20	28	Axial	0	2	0	0	465.94	455.85	10.1	467.10	1.16
29	29	Center	0	0	0	0	588.63	575.71	12.91	576.26	− 12.37
23	30	Axial	0	0	0	− 2	159.23	184.01	− 24.78	160.23	1.00

Variable	Code	− 2	− 1	0	1	2					
Temperature (°C)	X_1_	25	30	35	40	45					
Incubation time (days)	X_2_	2	3	4	5	6					
HAuCl_4_ conc. (µg/mL)	X_3_	200	400	600	800	1000					
Initial pH level	X_4_	4	5	6	7	8

According to the data that was collected, the values of the biosynthesized AuNPs ranged from a 159.23 to 866.29 µg/mL. The highest value of biosynthesized AuNPs (866.29 µg/mL) was obtained in the trial number 2 where the gold concentration was 1000 µg/mL, the pH was 6, the temperature was 35 °C, and the incubation time was 4 days. In contrast, the lowest value of biosynthesized AuNPs (159.23 µg/mL) was produced in run number 30 with gold concentration of 600 µg/mL, pH of 4, temperature of 35 °C, and incubation time of 4 days.

### Multiple regression analysis and the analysis of variance (ANOVA)

Table 2 displays the results of the analysis of variance (ANOVA) performed on the experimental data of CCD for the biosynthesis of AuNPs using the cell-free supernatant of *Streptomyces flavolimosus*. The model’s validity was assessed using the data recorded in Table 2, including adjusted R^2^ value, R^2^ value, predicted R^2^ value, *P*-value, lack of fit and *F*-value and the coefficient estimate values. In addition, the linear, interaction, and quadratic impacts of the four chosen process variables were evaluated^15^.

**Table 2:** Analysis of variance of CCD for AuNPs biosynthesis using *Streptomyces flavolimosus* cell-free supernatant as affected by temperature (°C), incubation period (days), HAuCl_4_ concentration (µg/mL) and initial pH level.

Source of variance		Coefficient estimate	Sum of squares	Degrees of freedom	Mean square	*F*-value	*P*-value
Model	Intercept	575.71	903,800	14	64,560.43	92.17	< 0.0001*
Linear effect	X_1_	20	9600.34	1	9600.34	13.71	0.0021*
	X_2_	− 39.19	36,859.26	1	36,859.26	52.62	< 0.0001*
	X_3_	152.55	558,500	1	558,500	797.39	< 0.0001*
	X_4_	47.37	53,847.92	1	53,847.92	76.88	< 0.0001*
Interaction effect	X_1_ X_2_	17.98	5173.71	1	5173.71	7.39	0.0159*
	X_1_ X_3_	43.36	30,080.84	1	30,080.84	42.94	< 0.0001*
	X_1_ X_4_	− 19.82	6287.27	1	6287.27	8.98	0.009*
	X_2_ X_3_	− 32.95	17,370.86	1	17,370.86	24.8	0.0002*
	X_2_ X_4_	37.35	22,323.96	1	22,323.96	31.87	< 0.0001*
	X_3_ X_4_	25.46	10,370.3	1	10,370.3	14.81	0.0016*
Quadratic effect	X_1_^2^	− 1.8	88.83	1	88.83	0.1268	0.7267
	X_2_^2^	− 10.37	2950.84	1	2950.84	4.21	0.058
	X_3_^2^	− 8.85	2150	1	2150	3.07	0.1002
	X_4_^2^	− 74.24	151,200	1	151,200	215.84	< 0.0001*
Error effect	Lack of fit		9360.18	10	936.02	4.08	0.067
	Pure error		1146.64	5	229.33		
R^2^	0.9885						
Adj R^2^	0.9778						
Pred R^2^	0.9392						
Adeq Precision	35.3418						

*F* Fishers’s function, *P* Level of significance.

*Significant values.

The determination coefficient (R^2^) was applied to measure the adequacy of the model since it quantifies the extent of response value variability that could be attributable to the experimental parameters. R^2^ values are always less than or equal to one, and when they are closer to one, it indicates that the model is more robust and can estimate the response more precisely. The model that had an R^2^ value that was more than 0.9 was regarded to have a very high correlation^68^. In the current study, the R^2^ value of the model used for the biosynthesized AuNPs is 0.9885 (Table 2), reflecting that 98.85% of variance in the biosynthesized AuNPs is attributable to the independent variables and just 1.15% of the total variance in the biosynthesized AuNPs is not explained by the independent factors. R^2^ value reflected a very good fit between the experimental and predicted values of the biosynthesized AuNPs. On the other hand, the regression model used for AuNPs biosynthesis has an adjusted determination coefficient (Adjusted R^2^) equal to 0.9778 (Table 2). Also, the high value of predicted R^2^ (0.9392) was in a reasonable agreement with adjusted R^2^ value which indicates that the model is good for predicting AuNPs biosynthesis to new experiments. The value of predicted R^2^ (0.9392) was in close agreement with the adjusted R^2^ value (0.9778). This demonstrated that there is a high compatibility between the observed and predicted values of the response^14^.

The value of adequate precision assesses the signal-to-noise ratio. A signal-to-noise ratio higher than 4 is desirable and reflective of the model’s precision^69^. In the present study, the model's adequate precision value was 35.34, indicating adequate design space for optimizing the biosynthesis of AuNPs at different values of the examined parameters.

In addition, the estimated coefficient demonstrated positive or negative effects on the biosynthesis of AuNPs. There are two types of interactions that can occur between two variables: antagonism (a negative coefficient) and synergism (a positive coefficient). If the estimated effect value is high, regardless of whether it is positive or negative, this indicates that the variable has a substantial impact on the response. On the other hand, if the effect is close to zero, this indicates that the variable has little or no influence on the response. If the coefficient of a tested variable has a positive sign, it indicates that yield increases as the variable’s value increases. Conversely, a negative sign indicates that yield increases at the low value of the variable^70^. If the coefficient for interactions between two variables is positive, this indicates that the interactions between the variables have a synergistic effect on the biosynthesis of AuNPs. The results indicated that the interaction effects between X_1_ X_2_, X_1_ X_3_, X_2_ X_4_, X_3_ X_4_ (Table 2) increase AuNPs biosynthesis. However, the presence of a negative coefficient indicates that the interactions between two factors have an antagonistic effect and decrease AuNPs biosynthesis. The results indicated that the interaction effects between X_1_ X_4_, X_2_ X_3_ decrease AuNPs biosynthesis.

*F-*values and probability values (*P-*values) (Table 2) were also estimated to determine the significance of each coefficient, which is essential for assessing the factors significance and for interpreting the pattern of their mutual interactions. Moreover, process factors with *P-*values ≤ 0.05 were considered to have significant effects on the response^70^. The results of the Fisher’s *F*-test (92.17), along with a very low *P*-value (< 0.0001), are presented in Table 2, and they reveal that the model is very significant. Based on the *P-*values of the coefficient, it can be concluded that linear effects of temperature (X_1_), incubation times in days (X_2_), HAuCl_4_ concentration ($\upmu$g/mL) (X_3_) and initial pH level (X_4_) are significant for AuNPs biosynthesis using the cell-free supernatant of *Streptomyces flavolimosus.* X_1_, X_2_, X_3_ and X_4_ had *F*-values of 13.71, 52.62, 797.39, and 76.88, and *P*-values of 0.0021, < 0.0001, < 0.0001, and < 0.0001; respectively. This highly significant that the four variables contribute as limiting variables. This indicates that the four variables contribute as limiting variables. Minor variations in their levels will affect AuNPs biosynthesis using *Streptomyces flavolimosus* cell-free supernatant. Furthermore, the *P-*values of the coefficient revealed that all interactions between the four tested factors were significant for AuNPs biosynthesis using *Streptomyces flavolimosus* cell-free supernatant.

The findings of the fit summary that are provided in Table 3 were used to determine which of the linear, 2FI, and quadratic models was the most appropriate model for the AuNPs biosynthesis using the cell-free supernatant of *Streptomyces flavolimosus*. The quadratic model is an appropriate model for the biosynthesis of AuNPs since the lack of fit is non-significant (*P*-value is 0.067 and the *F*-value is 4.08). Additionally, the R^2^ of 0.9885, the adjusted R^2^ value (0.9778), as well as the predicted R^2^ value (0.9392), are greater in the quadratic model in comparison to the values obtained in other models (Table 3). Quadratic model summary statistics for AuNPs biosynthesis showed a minimum standard deviation of 26.47. The predicted residual sum of squares (PRESS) demonstrates the validity of the model. If the PRESS statistic is low, the data points are well-fit by the model. The quadratic model achieves a minimum PRESS value of 55,565.78.

**Table 3:** Fit summary for of CCD for AuNPs biosynthesis using *Streptomyces flavolimosus* cell-free supernatant as affected by temperature (°C), incubation period (days), HAuCl_4_ concentration (µg/mL) and initial pH level.

Source	Sum of squares	*Df*	Mean square	*F-*value	*P-*value
Lack of fit tests					
Linear	254,400	20	12,718.18	55.46	0.0001*
2FI	162,800	14	11,625.48	50.69	0.0002*
Quadratic	9360.18	10	936.02	4.08	0.067

Source	Std. dev.	R^2^	Adjusted R^2^	Predicted R^2^	PRESS
Model summary statistics					
Linear	101.10	0.7206	0.6758	0.5828	381,500
2FI	92.88	0.8207	0.7264	0.7071	267,800
Quadratic	26.47	0.9885	0.9778	0.9392	55,565.78

*df* degree of freedom, two factors interaction: 2FI.

*Significant values.

By using multiple regression analysis to the experimental results, the following second-order polynomial equation was used to determine the predicted AuNPs biosynthesis (Y) that correspond to the optimum levels of initial pH, HAuCl_4_ concentration, incubation period, and temperature:

$${\text{Y}} = { 575}.{17 } + { 2}0{\text{ X}}_{{1}} {-}{ 39}.{\text{19 X}}_{{2}} + { 152}.{\text{55 X}}_{{3}} + { 47}.{\text{37 X}}_{{4}} + {17}.{\text{98 X}}_{{1}} {\text{X}}_{{2}} + { 43}.{\text{36 X}}_{{1}} {\text{X}}_{{3}} {-}{19}.{\text{82 X}}_{{1}} {\text{X}}_{{4}} {-}{ 32}.{\text{95 X}}_{{2}} {\text{X}}_{{3}} + { 37}.{\text{35 X}}_{{2}} {\text{X}}_{{4}} + { 25}.{\text{46 X}}_{{3}} {\text{X}}_{{4}} {-}{1}.{\text{8 X}}_{{1}}^{2} \, {-}{1}0.{\text{37 X}}_{{2}}^{2} \, {-}{8}.{\text{85 X}}_{{3}}^{2} \, {-}{74}.{\text{24 X}}_{{4}}^{2} .$$

Y is the predicted AuNPs biosynthesis, X_1_ (temperature), X_2_ (incubation period), X_3_ (HAuCl_4_ concentration) and X_4_ (initial pH).

### The model adequacy

The normal probability plot (NPP) is a metric that verifies the fitness of a model by indicating whether or not the residuals follow a normal distribution^23^. Residuals are differences between theoretical predictions and experimental findings; A low value means the model is adequate^71^. Figure 5B shows the NPP, which reveals that the residuals occur along the diagonal straight line of AuNPs biosynthesis. This illustrates that the predicted data well-fit with the experimental results, which verifies the accuracy of the model^58^.

Figure 5C compares the experimental results with predicted values for the biosynthesis of AuNPs by using the cell-free supernatant of *Streptomyces flavolimosus*. It demonstrates that all of the points are located very close to the prediction line, which suggests that the model adequately fits the experimental data^71^.

In addition, Fig. 5D shows Box–Cox plot of model transformation. It demonstrates that the red lines represent the low and high 95% confidence levels corresponding to the highest λ value (0.75 and 1.67; respectively). The blue line represents the current transformation (λ = 1) while the green line represents the best value of λ (λ = 0.73). Since the blue line lies between the two red lines, the model is in the optimal zone. This implies that no data transformation is needed, and the model is a good fit for the obtained experimental results^58^.

Figure 5E presents the externally studentized residuals against the values that were predicted for the AuNPs biosynthesis using the cell-free supernatant of *Streptomyces flavolimosus*. The graph revealed that the residuals are distributed randomly around the zero line, which suggests that the experimental results have a constant small variance^72^.

### Three dimensional (3D) plots

**Figure 6:**
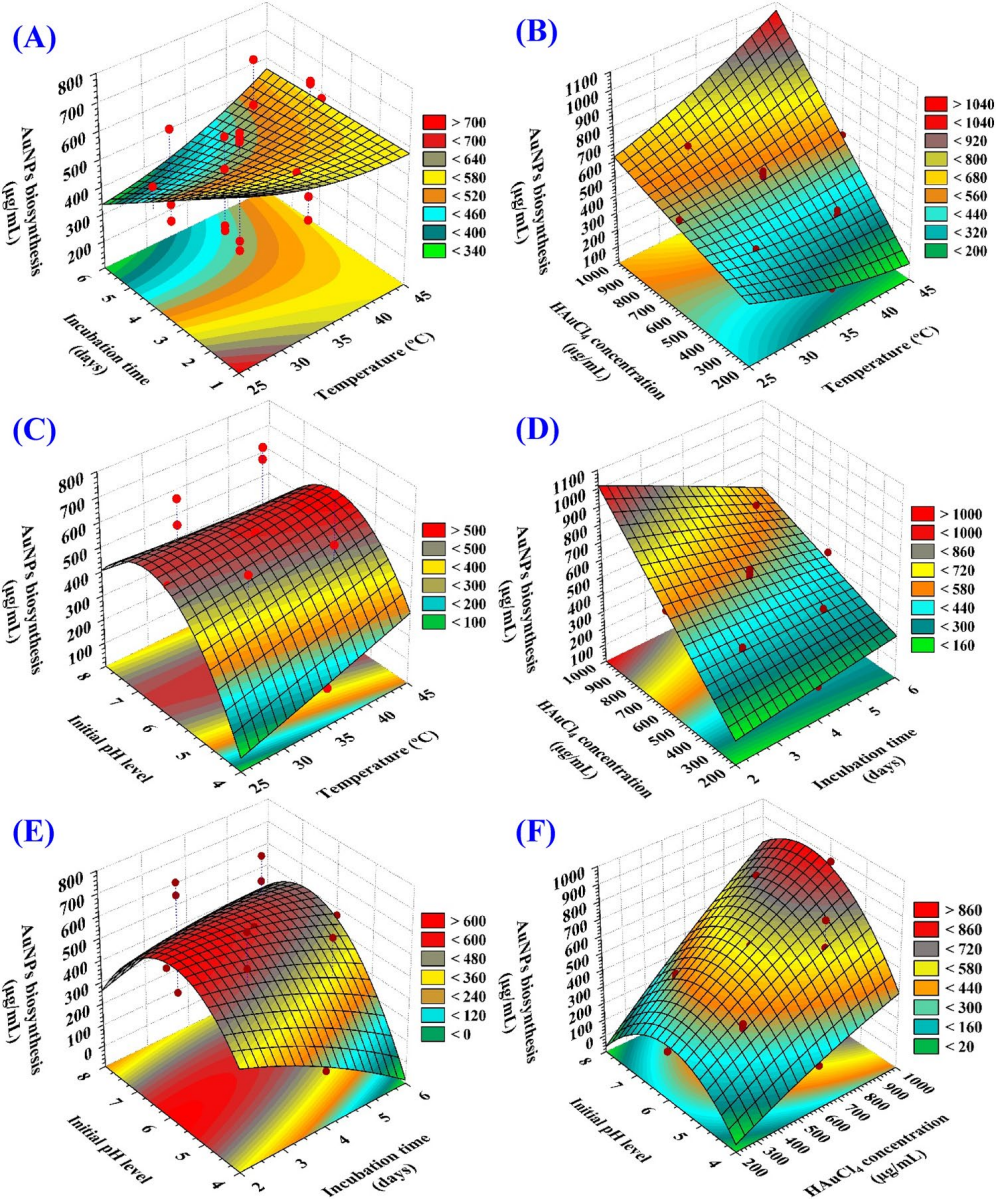
3D plots of the biosynthesized AuNPs using *Streptomyces flavolimosus* cell-free supernatant, showing the mutual interactions effects of the tested variables.

Figure 6A–F depicts the relationship between the biosynthesized AuNPs and the mutual interactions between studied variables to determine the optimum conditions for AuNPs biosynthesis using the cell-free supernatant of *Streptomyces flavolimosus*. For the pair-wise combinations of the four variables, 3D plots (X_1_ X_2_, X_1_ X_3_, X_1_ X_4_, X_2_ X_3_, X_2_ X_4_, X_3_ X_4_) were generated by plotting AuNPs biosynthesis on Z-axis against the X and Y axes for two independent variables while keeping the values of the other two variables at their center points.

The 3D plot (Fig. 6A) demonstrating the mutual interactions of the temperature (X_1_) and the incubation time (X_2_) on the biosynthesized AuNPs, while HAuCl_4_ concentration (X_3_), and initial pH level (X_4_) were maintained at their central levels. Figure 6A demonstrates that decreased levels of both temperature (X_1_) and incubation time (X_2_) support the highest AuNPs production, while the AuNPs biosynthesis decrease gradually with increasing temperature or incubation time.

### The effect of temperature on AuNPs biosynthesis

In the current study, the optimal temperature for maximum gold nanoparticles biosynthesis was 35 °C. Kulkarni et al.^73^ observed that the nanoparticles formation rate and, consequently, the nanoparticles size could be controlled by adjusting parameters such as gold concentration, temperature, pH, and exposure time to HAuCl_4_. Camas et al.^74^ showed that the optimal temperature for maximum gold nanoparticles biosynthesis was 35 °C and gold nanoparticles biosynthesis is negatively impacted by an increase in temperature. The optimal temperature for maximum gold nanoparticles biosynthesis by *Streptomyces* sp. ERI-3 supernatant was 30 °C^75^.

### The effect of incubation time on AuNPs biosynthesis

In the current study, the optimal incubation time for maximum gold nanoparticles biosynthesis was 4 days. Zonooz et al.^75^ demonstrated that higher yield of gold nanoparticles by *Streptomyces* sp. ERI-3 supernatant was produced after 96 h of incubation. On the other hand, Camas et al.^74^ demonstrated that the maximum gold nanoparticles biosynthesis by *Citricoccus* sp. K1D109 was produced after 24 h of incubation, and that the biosynthesis decreases as the interaction time increases.

Figure 6B describes the relationship between the biosynthesized AuNPs and the mutual interactions between the temperature (X_1_) and the HAuCl_4_ concentration (X_3_), while incubation time (X_2_) and initial pH level (X_4_) were maintained at their central levels. It can be seen that the biosynthesized AuNPs increased gradually by increasing the temperature to 35 °C and the HAuCl_4_ concentration near to (1000 µg/mL).

### The effect of HAuCl_4_ concentration on AuNPs biosynthesis

With increasing gold chloride concentration, the biosynthesis of AuNPs was seen to increase. It could be attributable to the high concentration of gold chloride, where more Au^+3^ ions were available for reduction to AuNPs^76^. Zonooz et al.^75^ stated that the high titer of AuNPs biosynthesis by *Streptomyces* sp. ERI-3 supernatant (1450 a.u.) was achieved with a solution of 3 mM HAuCl_4_. However, the synthesis of AuNPs was reduced at concentrations of 3.5 and 4 mM, mostly probably because of the toxicity of metal ions on components involved in the synthesis of AuNPs.

Figure 6C highlights the relationship between the biosynthesized AuNPs and the mutual interactions between the temperature (X_1_) and initial pH level (X_4_), while incubation time (X_2_) and HAuCl4 concentration (X_3_) were maintained at their central levels. The biosynthesized AuNPs value increased until the optimal pH (at 6) was reached, and then reduced when the pH level rose above that. By increasing temperature, the biosynthesized AuNPs value increased up to 35 °C. However, subsequent increases in temperature did not affect the AuNPs' biosynthesis in any significant manner.

### Effect of pH on AuNPs biosynthesis

In the current study, the optimal pH for maximum AuNPs biosynthesis was 6. Zonooz et al.^75^ stated that the optimal pH for maximum AuNPs biosynthesis by the supernatant of *Streptomyces* sp. ERI-3 supernatant was 6. The maximum AuNPs biosynthesis by microbial cells typically takes place in the pH range of 2 to 6, and variations in pH could have an effect on the size distribution of AuNPs^77^. Changing the reaction mixture pH from 3 to 9 caused a range of colours in the aqueous solution, including dark pink, light pink, orange, dark purple, red, greenish-blue and green^75^. Shakouri et al.^78^ reported that by altering the pH values of the reaction mixture from 2 to 6, a variety of aqueous solution colors including very dark purple, light red, greenish blue, light green and dark green was resulted. The increase in colour intensity of the reaction mixture was due to the increasing number of nanoparticles formed by *Aspergillus flavus* supernatant as the result of reduction of gold ions present in the aqueous solution. Saha et al.^76^ stated that the biosynthesis of AuNPs was maximized when the pH was acidic, whereas at alkaline pH only slight variations were observed in AuNPs biosynthesis.

It was established that the pH was a key parameter that affected the synthesis of gold nanoparticles. The size, shape, and number of particles were all affected by changes in pH of the substrate or medium^77^. The gradual increase in pH accelerates the reduction process, which results in the formation of tiny AuNPs^79^. AuNPs biosynthesis by *Aspergillus terreus* is regulated by changes in pH. When pH is adjusted to 8, the shape of AuNPs shifts from spherical to rod-like, and their size ranges from 20 to 29 nm. The synthesized AuNPs have spherical shape when the pH is set to 10, and their average size ranges from 10 to 19 nm^80^.

Figure 6D describes the relationship between the biosynthesized AuNPs and the mutual interactions between the incubation time (X_2_) and HAuCl_4_ concentration (X_3_), while temperature (X_1_) and initial pH level (X_4_) were maintained at their central levels. It is evident that the amount of biosynthesized AuNPs rises gradually as the incubation time as well as the concentration of HAuCl_4_ were increased.

Figure 6E explains the relationship between the biosynthesized AuNPs and the mutual interactions between incubation time (X_2_) and initial pH (X_4_), while keeping the temperature (X_1_) and HAuCl_4_ concentration (X_3_) at their central levels. By Fig. 6E analysis, it was found that the maximum biosynthesized AuNPs will obtain when the initial pH level 6 and 4 days of incubation time were used. Decreasing or increasing the pH value or increase the incubation time, the AuNPs will be decreased.

Figure 6F describes the relationship between the biosynthesized AuNPs and the mutual interactions between HAuCl_4_ concentration (X_3_) and initial pH (X_4_) while keeping the temperature (X_1_) and incubation time (X_2_) at their central levels. It reveals when pH level is at its optimum (at 6), the maximum biosynthesized AuNPs was achieved. The HAuCl_4_ concentration controls the AuNPs production. Increasing the HAuCl_4_ concentration, leads to the highest amount of AuNPs biosynthesis. Vice versa, when the HAuCl_4_ concentration is decreased, the lowest amount of AuNPs is produced.

### Artificial neural network (ANN) model for prediction of AuNPs biosynthesis

The artificial intelligence-based approach was employed for analyzing, validating and predicting AuNPs biosynthesis using the cell-free supernatant of *Streptomyces flavolimosus* biomass (Table 1). The ANN is an advanced technique in artificial intelligence that instructs computers to develop reliable and efficient models, to analyze and interpret data in a manner comparable to that of the human brain. The ANN is a machine learning technique that builds an adaptable framework that enables computers to gain knowledge from their previous mistakes and continuously improve. The construction or topology of artificial neural networks is regulated by two principal parameters including the number of layers and the number of nodes or neurons in each hidden layer. The network design of ANN modelling comprises learning and training processes, validation and verification of the final ANN model.

**Figure 7:**
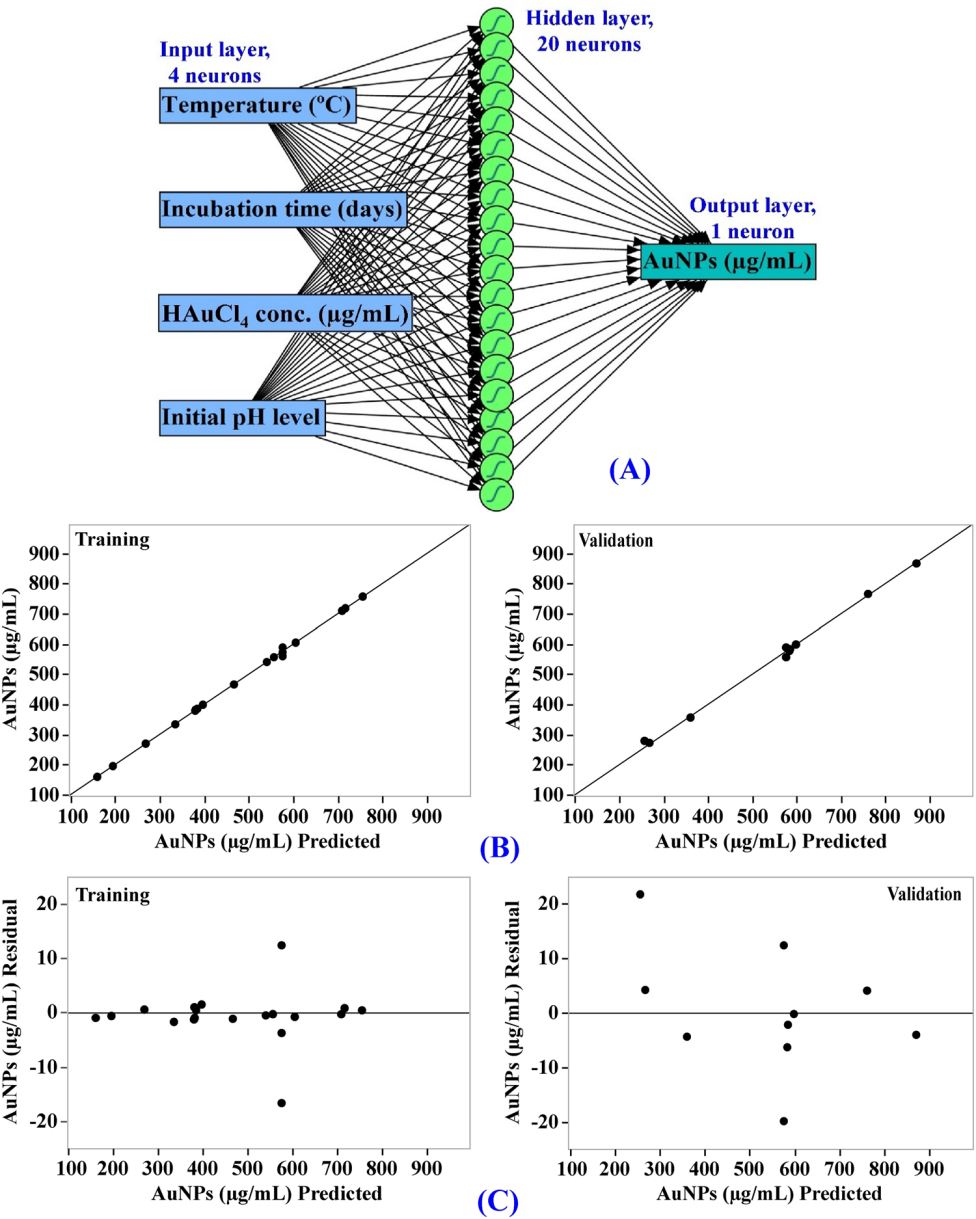
The final artificial neural network of the biosynthesized AuNPs (**A**), the ANN predicted versus actual (**B**), and the residuals versus ANN predicted (**C**) values of the biosynthesized AuNPs using *Streptomyces flavolimosus* cell-free supernatant.

Input neuron network topology was used to build an ANN architecture for optimizing AuNPs biosynthesis using the cell-free supernatant of *Streptomyces flavolimosus* biomass. Simple neural network architecture has interconnected artificial neurons in three layers including output layer, hidden layers and input layer. In this study, one input layer comprised of the four independent factors (temperature (°C), incubation period (days), HAuCl_4_ concentration (µg/mL) and initial pH level) enters the artificial neural network. Input nodes process the data, analyze or categorize it, and pass it to the subsequent layer. One output layer provides the final outcome of the artificial neural network's data processing (AuNPs biosynthesis, µg/mL) (Fig. 7A). The optimal ANN parameters were adjusted to number of TanH nodes in the first layer (20), a validation method (holdback proportion, 0.2), a learning rate of 0.1, number of tours (2000), and fitting option was transforming covariates.

### Evaluation of ANN model

The values predicted AuNPs biosynthesis by ANN corresponding to each experimental result were given in Table 1. The predicted values of AuNPs biosynthesis by ANN were drawn against the actual values (Fig. 7B). In both the training and validation phases, the points are gathered closer to the line showing the optimal prediction, indicating that the model is reliable. The scattering of residual data above and below the regression line revealed a normal scattering of the residuals (Fig. 7C) which support for the suitability of the ANN model.

### Comparison of prediction potential of ANN versus CCD

As illustrated in Table 1, the AuNPs biosynthesis values predicted by ANN exhibit a more reasonable agreement with the experimental result, and the residuals values were lower than those obtained by the CCD model. The model’s performance was compared using the model comparison dialog in the JMP Pro16. For comparison, several error functions, as well as the R^2^ used to evaluate the prediction ability of the CCD and ANN. The most frequently employed functions for the comparison were R^2^, average absolute error (AAE) and root average squared error (RASE) for each regression model which are shown in Table 4. The prediction ability of the CCD and ANN was compared, the higher value of R^2^ (0.9981) along with lower value for RASE (7.64) and lower value for AAE (4.60) (Table 4) confirms ANN as the best model with a higher predictive capacity for the optimal levels of different physicochemical variables for AuNPs biosynthesis. This finding can be explained by the capability of ANN to give good performance, as training of the neurons was repeated multiple times for various physicochemical variables.

**Table 4:** ANN analysis and modeling comparison of predictive capability between CCD and ANN for AuNPs biosynthesis using *Streptomyces flavolimosus* cell-free supernatant as affected by temperature (°C), incubation period (days), HAuCl_4_ concentration (µg/mL) and initial pH level.

Measure	ANN		Overall model performance		
	Training	Validation	Statistics	Measures of fit for CCD	Measures of fit for ANN
R^2^	0.9989	0.9967	R^2^	0.9885	0.9981
RASE	5.54	10.67	RASE	18.72	7.64
MAD	2.93	7.95	AAE	15.89	4.60
SSE	612.82	1138.01	Freq	30	30
Sum freq	20	10			

*MAD* mean absolute deviation, *SSE* the sum of squares error, *RASE* root average squared error, *AAE* average absolute error for each model.

In the laboratory, the CCD and ANN models for maximizing AuNPs biosynthesis using *Streptomyces flavolimosus* cell-free supernatant were validated using the estimated predicted values of the four tested variables. In order to validate both CCD and ANN models, the optimal estimated predicted values that maximize AuNPs biosynthesis using *Streptomyces flavolimosus* cell-free supernatant were calculated to be a concentration of HAuCl_4_ that was 1000 μg/mL, an initial pH of 6, a temperature of 35 °C, and an incubation time of 4 days. Based on CCD and ANN data analysis, the theoretical predicted AuNPs biosynthesis was 845.4 μg/mL and 870.36 μg/mL, respectively. The theoretical predicted values were assessed experimentally. Under these conditions, the maximum experimental value of AuNPs biosynthesis using *Streptomyces flavolimosus* cell-free supernatant was 866.29 μg/mL. The verification showed a high precision degree of both models. However, the theoretical predicted value of AuNPs biosynthesis by ANN (870.36 μg/mL) was considerably closer to the experimental value (866.29 μg/mL) than that predicted by CCD (845.4 μg/mL), which indicates that ANN has stronger prediction potential in comparison to the CCD.

### Desirability function (DF)

**Figure 8:**
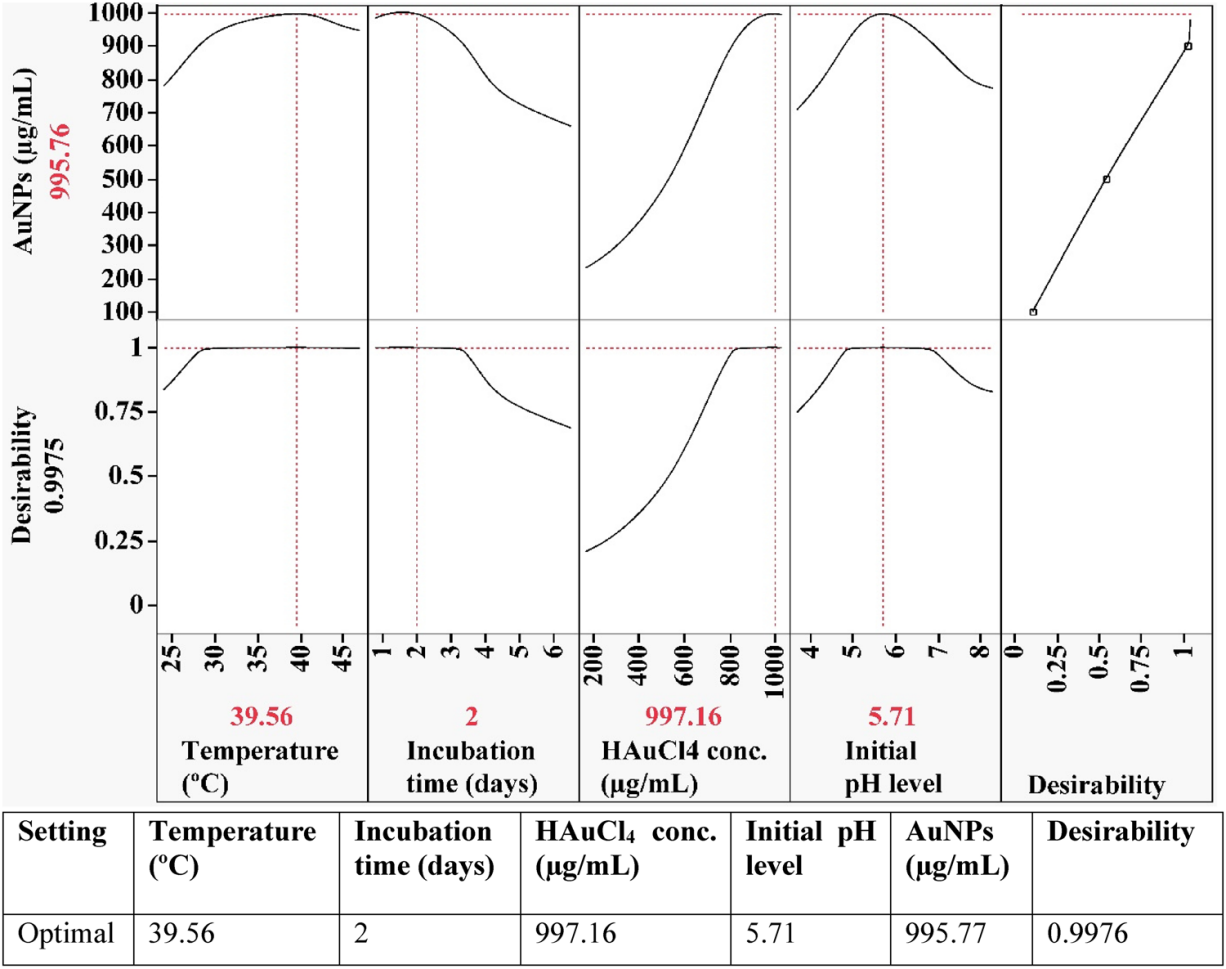
The optimization plot displays the desirability function and the optimum predicted values of the biosynthesized AuNPs using *Streptomyces flavolimosus* cell-free supernatant.

The desirability function, shown in Fig. 8, was performed in order to find the optimal predicted conditions for biosynthesis of gold nanoparticles that would yield the highest value.

The software Design Expert’s desirability function is set for any factor from 0 (undesirable) to 1 (desirable)^69^. Generally, the value of the desirability function is estimated theoretically before experimental verification of the optimization process. The maximum value of gold nanoparticles biosynthesis (995.77 µg/mL) using the cell-free supernatant of *Streptomyces flavolimosus* was found under the optimum conditions: HAuCl_4_ concentration (997.16 µg/mL), initial pH level 5.71, temperature 39.56 °C and incubation time 2 days. Under these conditions, the maximum value of gold nanoparticles biosynthesis using the cell-free supernatant of *Streptomyces flavolimosus* (900.53 µg/mL) was confirmed, and the results were compared to the value that had been predicted by the polynomial model (995.77 µg/mL). The validation indicated a high level of model accuracy, demonstrating the model’s validity at the factor levels applied.

### Evaluation of cytotoxicity and antitumor activity in vitro

**Figure 9:**
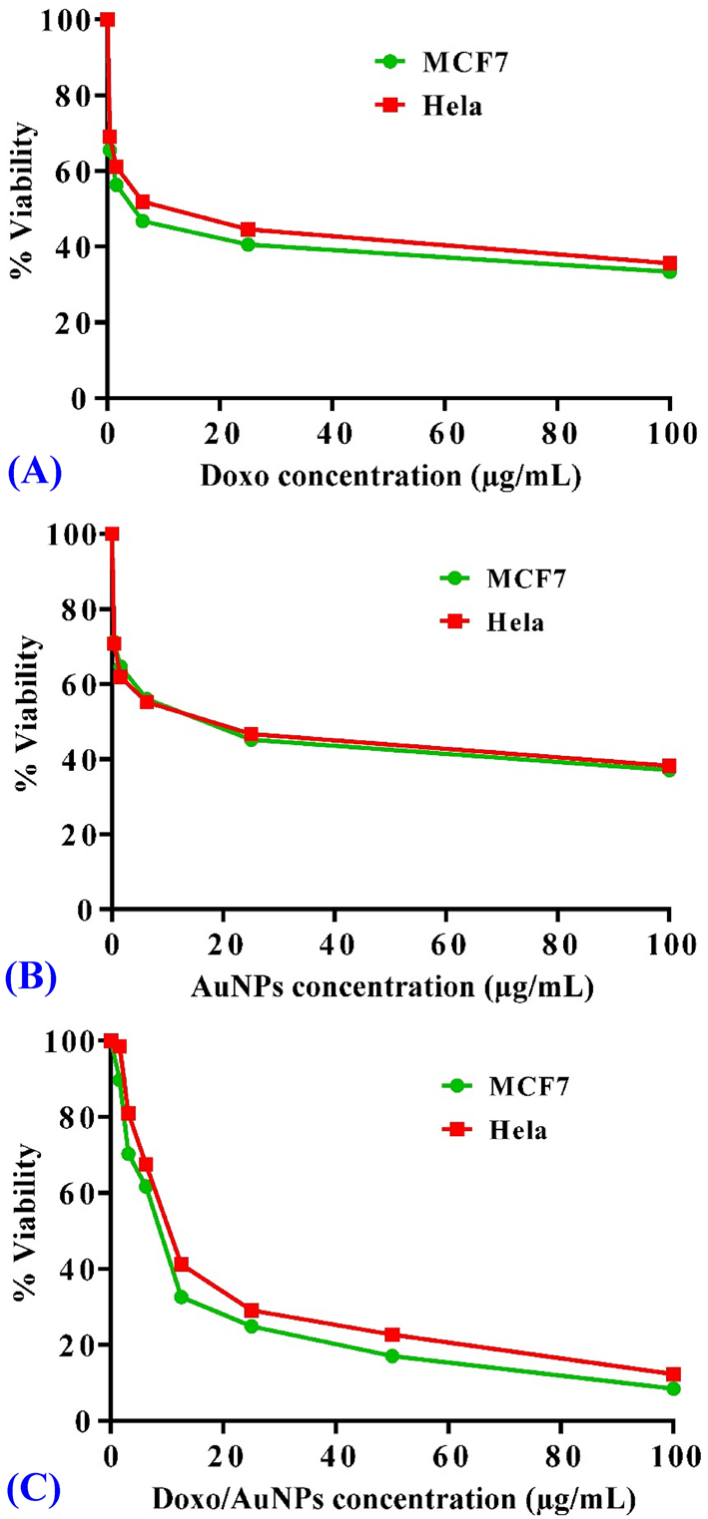
Cytotoxicity of AuNPs in breast cancer (MCF-7) and cervical epithelioid carcinoma (Hela) cell line. Concentration-response plots of treatment with Doxo (positive control) in MCF-7 and Hela cell lines (**A**), concentration-response plots of treatment with AuNPs in MCF-7 and Hela cell lines (**B**), and concentration-response plots of treatment with Doxo/AuNPs combination in MCF-7 and Hela cell lines (**C**). The experiment was repeated three independent times. Data are expressed as means ± SEM.

The anticancer efficacy (in vitro) of AuNPs synthesized by *Streptomyces flavolimosus* was evaluated compared to Doxorubicin (Doxo) using MTT assay against two human cancer cell lines: cervical epithelioid carcinoma (Hela) and breast cancer (MCF-7). Doxo significantly inhibited cell viability (Fig. 9A) in MCF-7 and Hela cells, with IC_50_ value of 4.84 ± 0.28 μg/mL and 9.41 ± 0.31 μg/mL in MCF-7 and Hela cells, respectively. The obtained results demonstrated that AuNPs exhibited a cytotoxic potential on the tested cell lines (Fig. 9B). In addition, treatments based on AuNPs can destroy cancer cells with an IC_50_ value of 13.4 ± 0.44 μg/mL and 13.8 ± 0.45 μg/mL in MCF-7 and Hela cells, respectively. Moreover, Doxo and AuNPs combination significantly inhibited cell viability in MCF-7 and Hela cells, with IC_50_ value of 8.32 ± 0.6 μg/mL and 11.72 ± 0.9 μg/mL in MCF-7 and Hela cells, respectively. Accordingly, addition of Doxo to AuNPs decreased significantly IC_50_ of AuNPs from 13.4 ± 0.44 μg/mL and 13.8 ± 0.45 μg/mL to 8.32 ± 0.6 μg/mL and 11.72 ± 0.9 μg/mL in MCF-7 and Hela cells; respectively. Thus, the combination of Doxo and AuNPs increased the anticancer potential of AuNPs supporting the additive impact of the anticancer potential when AuNPs and Doxo were used in combination. The rational of using Doxo and AuNPs in combination is that the dose of each agent can be reduced to give the same required effect as well as minimizing the side effects of Doxo. These results suggest that AuNPs have the potential to be used as an anticancer agent. Virmani et al*.*^81^ investigates the anticancer activity of biogenic and chemically produced AuNPs in relation to malignant and normal cell lines. They observed that the biologically produced AuNPs greatly suppressed the proliferation of malignant cells in a dose-dependent manner with an IC_50_ value of approximately 200 µg/mL. On the other hand, it was demonstrated that AuNPs are not toxic to normal cells. Myco-synthesized AuNPs made with the aqueous extract of the endophytic fungus *Cladosporium* sp. demonstrated anti-cancer efficacy against MCF-7 by the induction of the apoptotic pathway^82^. IC_50_ value was determined to 38.23 µg/mL. The study by Datkhile et al*.*^83^ evaluates the anticancer activities of AuNPs that were biologically produced by employing the extract of *Nothapodytes foetida*. The findings of the cytotoxicity test demonstrated that the AuNPs had significant cytotoxic activity against cancer cells; the IC_50_ value were 5.28, 7.2, and9.67 μg/mL for HCT-15, HeLa, and MCF-7 tumor cells, respectively.

The anti-cancer effects of AuNPs produced by Kaempferol were tested on the MCF-7 cancer cell line. The results showed a dose- and time-dependent reduction in the viability of MCF-7 breast cancer cells^84^. According to Kalaivani et al*.*^85^, AuNPs biosynthesized using chitosan exhibited cytotoxic activity against MCF-7 cell lines with an IC_50_ value of 250 μg/mL. Clarance et al*.*^86^ investigates the anticancer potentials of the AuNPs that were produced by a green synthesis approach with an endophytic strain of *Fusarium solani*. They found that AuNPs displayed a dose-dependent cytotoxic effect on MCF-7 and HeLa cells. IC_50_ value was determined to be 0.8 ± 0.5 μg/mL and 1.3 ± 0.5 μg/mL on MCF-7 and HeLa cell lines, respectively. Asl et al*.*^87^ investigate the antitumor activity of biogenically produced AuNPs on cisplatin-resistant A2780CP ovarian carcinoma cells. In a time- and dose-dependent manner, AuNPs suppressed the proliferation of cisplatin-resistant ovarian carcinoma cells. Also, Abdulateef et al.^88^ investigate the cytotoxic effect of the AuNPs synthesized using bovine serum albumin and pulsed laser ablation technique in simulated body fluid. They found that the treatment had no effect on normal fibroblast cells. However, a dose-dependent inhibition effect is observed on the growth of cervix cancer cells (HeLa).

Multiple studies have demonstrated that the cytotoxicity of AuNPs is caused by the induction of oxidative stress. HeLa cervical cancer cells displayed enhanced reactive oxygen species (ROS) generation and oxidative stress when exposed to 1.4 nm AuNPs, resulting in the oxidation of proteins and lipids, a severe impairment of the mitochondrial function, and ultimately cell death^89^. Dickson et al.^90^ investigate the cytotoxic effect of AuNPs that were produced via green sources on HeLa and melanoma tumor cells. The results showed that the cytotoxicity was dose-dependent and the impact of AuNPs on cell viability were caused by the induction of apoptosis.

The effect of AuNPs and doxorubicin together on cell viability on MCF-7 and Hela cells was checked. The results showed that Doxo and AuNPs combination significantly inhibited cell viability in MCF-7 and Hela cells, with IC_50_ value of 17.71 ± 1.2 μg/mL and 23.11 ± 1.7 μg/mL in MCF-7 and Hela cells, respectively. Thus, this can be understood as an additive impact of the anticancer potential when AuNPs and Doxo were used in combination. The concentration-response of treatment with Doxo/AuNPs combination in MCF-7 and Hela cell lines is shown in the Fig. 9C.

Aboyewa et al*.*^91^ examined the cytotoxicity of the co-treatment in MDA-321, KMST-6, HT-29, HeLa and Caski cell lines. They investigated the mechanistic effects of cytotoxicity induced by the co-treatment of Caco-2 cells with biogenic AuNPs and DOX. Aboyewa et al*.*^91^ suggest that these biogenic AuNPs can serve as drug sensitizers when used in combination with chemotherapeutic drugs and enhance their efficacy at a very low dosage. They reported that co-treatment of Caco-2 cells with DOX and the biogenic AuNPs enhances the drugs' effects and efficacy through a synergistic anti-cancer mechanism. Additionally, they reported that the biogenic AuNPs were effectively taken up by the cancer cells, which, in turn, may have enhanced the sensitivity of Caco-2 cells to DOX. Moreover, the combination of DOX and the biogenic AuNPs inhibited the long-term survival of Caco-2 cells, reduced the production of reactive oxygen species (ROS), induced apoptosis, increased mitochondrial depolarization and caused a rapid depletion of ATP levels.

### Assessment of antitumor activity of AuNPs in vivo in EAC model

Following the efficient induction of Ehrlich solid tumors and once the tumor volume reached 100–200 mm^3^, the mice were randomly divided into four groups to receive therapy for 21 days. Mice bearing solid tumors were treated with AuNPs (5 mg/kg/day) as single therapy or in combination with Doxo.

### Influence of AuNPs on tumor volume in mice carrying EAC

**Figure 10:**
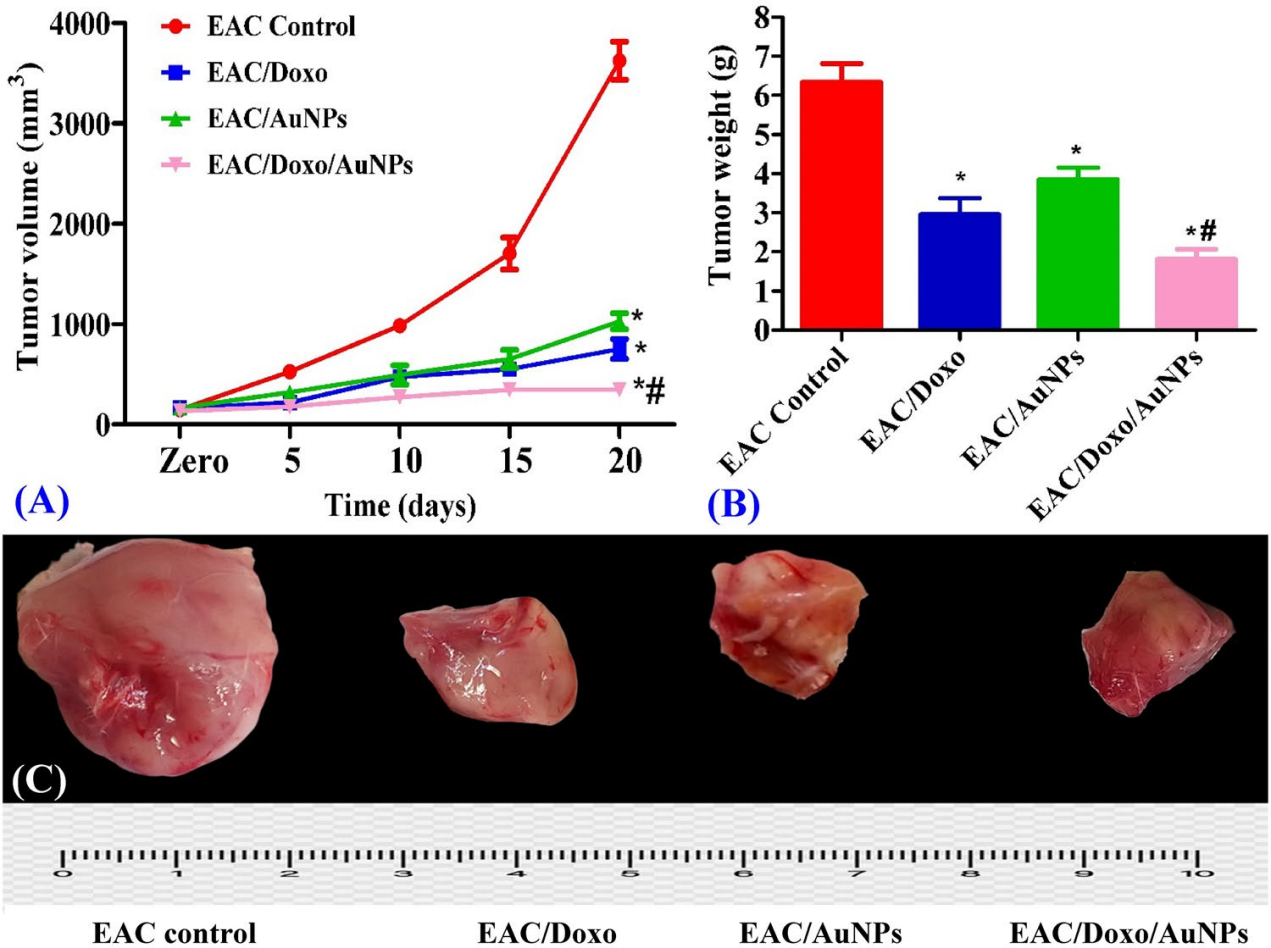
Effects of AuNPs alone or in combination with Doxo on tumor volume (nm^3^) (**A**) and tumor weight (g) (**B**) of EAC bearing mice. All tumor images (**C**) were taken at the same magnification power, zooming and distance from camera. Data are expressed as means ± SEM (n = 6 for EAC control group and n = 7 for EAC/Doxo, EAC/AuNPs, and EAC/Doxo/AuNPs groups). Statistical comparisons were made using one way of analysis of variance (ANOVA) followed by Tukey–Kramer post hoc test for multiple comparisons. *Indicates significant difference vs. EAC control at *P* < 0.05; ^#^represents significant difference vs. EAC/AuNPs at *P* < 0.05.

Evaluation of tumor volume every five days during the experiment demonstrated a significant decline in tumor growth. At the end of the experiment, the average tumor volume in the EAC control mice had increased from 145.6 to 3623 mm^3^. After 21 days of treatment, in comparison to EAC control mice, the mice that received Doxo and AuNPs treatment showed tumor growth inhibition rate of 79.2% and 71.6%; respectively; indicating a significant antitumor activity of AuNPs in EAC bearing mice. While treatment with a combination of Doxo and AuNPs resulted in a 90.5% tumor growth inhibition rate compared to EAC control group after 21 days of treatment suggesting that treatment with both Doxo and AuNPs at the same time was more effective at reducing tumor volume than treatment with either drug alone (Fig. 10A).

### Influence of AuNPs on tumor weight in mice carrying EAC

Assessment of Ehrlich carcinoma weight during the experiment demonstrated a significant decline in tumor weight. After 21 days of treatment, in comparison to EAC control mice, the mice that received Doxo and AuNPs treatment showed that the tumor weight decreased by 53.33% and 39.29%; respectively; indicating a significant antitumor activity of AuNPs in EAC bearing mice. In comparison, therapy with a combination of AuNPs and doxorubicin led to a decrease in tumor weight that was 71.39% compared to that of the EAC control group (Fig. 10B,C). This significant tumor suppression in the group that was treated with the combination of AuNPs and doxorubicin demonstrates that the anticancer efficacy of Doxo is significantly increased when combined with AuNPs.

In such areas of study, the application of AuNPs is attracting interest owing to their unique characteristics compared to the very toxic NPs. AuNPs have excellent conductivity, size-dependent characteristics, optical characteristics, low toxicity, simplicity, and functional diversity. AuNPs are considered to be relatively biologically non-reactive and therefore suitable for in vivo applications. The strong optical characteristics of light absorbance of AuNPs due to localized surface plasmon resonance, enhanced permeability and retention in tumor tissue, their interaction with radiation to produce secondary electrons, as well as their capacity to be conjugated with drugs or other medications are also advantageous^88,92,93^. Due to the surface plasmon resonance characteristic, laser stimulation of AuNPs induces hyperthermia and thermal damage of the tumor tissue while causing minimal harm to normal tissue^93^. Myco-synthesized AuNPs made with the aqueous extract of the endophytic fungus *Cladosporium* sp. demonstrated strong cytotoxic efficacy against the progression of tumors in mice models carrying the cancerous tissue. Myco-synthesized AuNPs significantly reduced the body weight, ascites volume, and increased the lifespan of EAC bearing mice^82^.

### Influence of AuNPs on histopathological findings in EAC bearing mice

**Figure 11:**
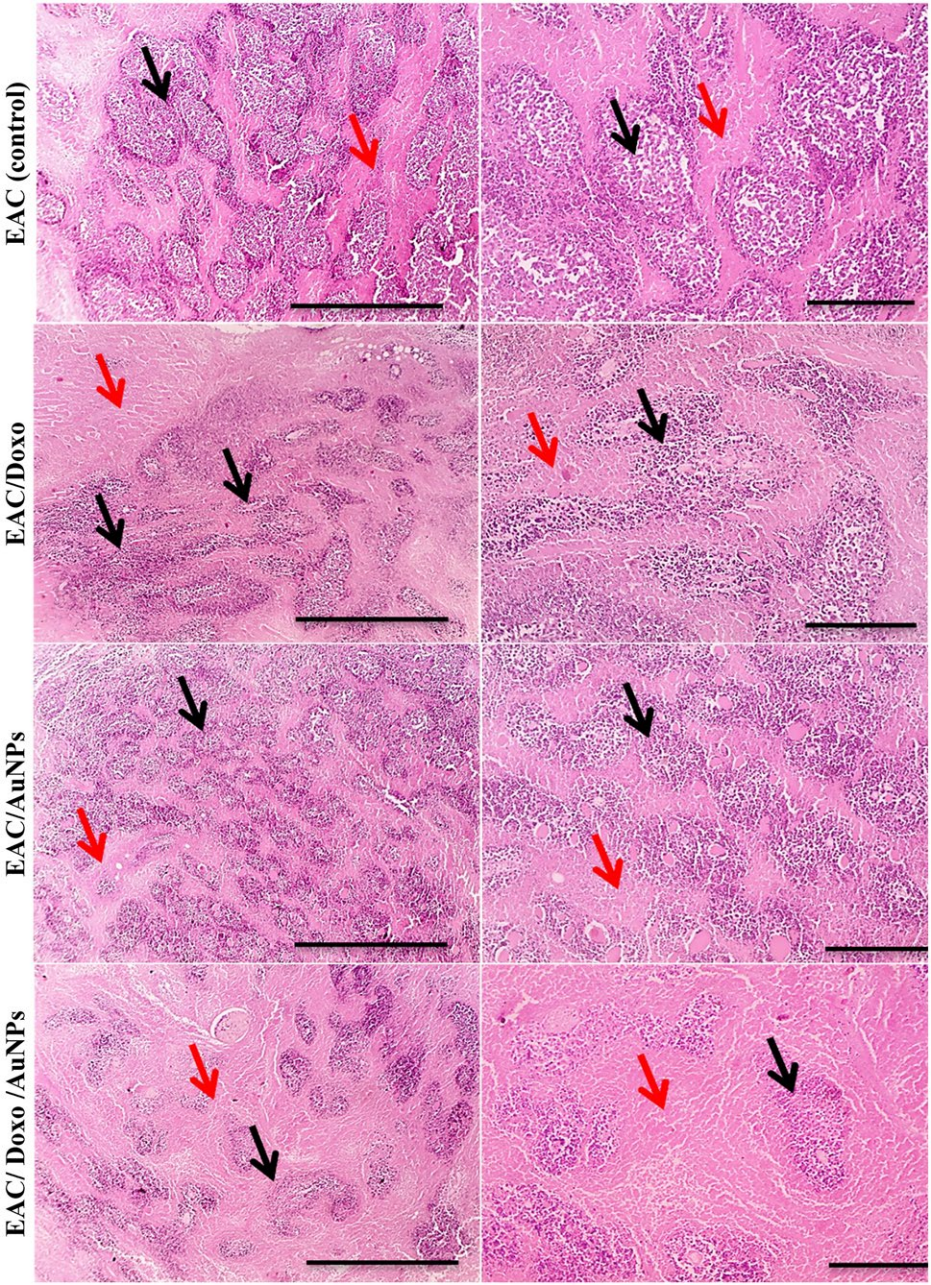
Microscopic pictures of H&E-stained sections from tumor masses showing lower viable foci consisting of large, round and polygonal deeply stained tumor cells (black arrows) interspaced by increased eosinophilic necrotic zones (red arrows) in treated groups with Doxo, AuNPs and combination of Doxo and AuNPs when compared with untreated EAC. A marked reduction in tumor growth was observed in group treated with combination of Doxo and AuNPs. Magnifications × 40: bar 20 and × 100: bar 100.

Microscopic pictures of Hematoxylin and Eosin-stained sample from EAC control mice showing numerous viable foci consisting of large, round and polygonal deeply stained tumor cells (black arrows) interspaced by small eosinophilic necrotic zones (white arrows) in subcutaneous and muscular tissues (Fig. 11). Number of viable foci (black arrows) and number of viable tumor cells are moderately decreased associated with moderately increased size of necrotic areas in group treated with Doxo. Number of viable foci (black arrows), number of viable tumor cells are slightly decreased with conversely increased size of necrotic areas (white arrows) in group treated with AuNPs. Number of viable foci (black arrows) and number of viable tumor cells are markedly decreased associated with markedly increased size of necrotic areas in group treated with a combination of Doxo and AuNPs.

Because AuNPs are a good candidate for use in biomedical applications for photothermal therapy, biosensors, cancer treatment, gene delivery, drug delivery, and imaging techniques, it is essential to evaluate their behavior in an animal model. Several studies indicate that AuNPs accumulate in certain organs of rats^94^. Some of the factors that influence the toxicity of AuNPs include physical dimensions such as their size, dose, and shape as well as the length of exposure, their metabolism, surface chemistry, surface charge, composition, cell type and immune response to the AuNPs^95,96^. The nanoparticles administration method may also affect toxicity, the injection of the nanoparticles through tail vein was less toxic than intraperitoneal and oral administration^96^. Additionally, the production method (chemical, physical, and biological methods) of the nanoparticles could be a significant factor in the toxicity effects of the nanoparticles^94^.

Yahyaei et al*.*^94^ evaluate the influence of the biologically prepared 50–70 nm AuNPs in vitro and in vivo in intraperitoneally injected rats. MTT assay results revealed that AuNPs had somehow toxic effects which depend on their doses. The in vitro and in vivo behaviors of the produced AuNPs were different and AuNPs even in high concentration induced low changes in the rat organs. This may be due to the short exposure and the use of the biologically produced AuNPs. Shukla et al.^97^ reported that low concentrations of gold nanoparticles do not result in a noticeable reduction in body weight or substantial toxicity. Red blood cells, hematocrit, and body weight all decreased in high gold nanoparticle concentrations. Yah^98^ reported that AuNPs with a diameter of approximately 18 nm can enter cells without causing cell damage or toxicity. Consequently, the small size of the AuNPs plays a significant role and facilitates their incorporation into biological systems for subsequent probing and modification. Some exhibit toxicity due to the presence of surface-coated ligands, while others, due to their large surface-to-volume ratio, serve as platforms for increasing surface particle activity. Furthermore, it has been demonstrated that the influence of AuNP toxicity varies based on particle shape. Among the shapes, it has been reported that rod-shaped AuNPs are more toxic than their spherical counterparts. There is some information regarding the size-dependent elimination of chemically produced AuNPs from animal blood. For instance, it was reported that AuNPs with sizes of approximately 18 nm were removed from the bloodstream and accumulated in the spleen and liver^99^. In contrast to the larger AuNPs (50–100 nm), the smaller ones (5–15 nm) can easily be distributed throughout the rat organs^100^. Yahyaei et al*.*^94^ demonstrated that short-term exposure to AuNPs had little effect on the liver and kidneys. On the other hand, short-term exposure to chemically produced AuNPs (50 nm) had negligible effects on liver enzymes and no effects at all on kidney enzymes^101^.

To avoid accumulation and subsequent toxicity, nanoparticles (NPs) must be metabolized or eliminated from the body. The liver and the kidneys are the two primary pathways through which NPs are eliminated from the body. The mononuclear phagocyte system or tissue-resident phagocytes catch NPs in the hepatic pathway, leading to their accumulation in the liver and spleen. The NPs are then subjected to biliary clearance and excretion via faeces and renal filtration route and eliminated through the urine^102^. At the cellular level, it was demonstrated that AuNPs uptake occurred via receptor-mediated endocytosis, with maximum uptake occurring when nanoparticle sizes were approximately 50 nm^103^. Hainfeld et al.^104^ demonstrated that small-sized AuNPs can cross the blood–brain barrier, and that this penetration can be controlled by ion channel blockers. They demonstrated that there were no adverse effects on the brains of the animals and suggested using AuNPs for drug delivery purposes.

## Conclusion

The current study describes a biological-based, eco-friendly, and cost-effective approach for AuNPs green synthesis using the cell-free supernatant of *Streptomyces flavolimosus* as a novel source for AuNPs biosynthesis. Various bioprocess parameters were optimized and AuNPs biosynthesis was maximized using a mathematical models, central composite design and artificial neural network. Results of this study confirmed the efficacy of artificial neural networks in the precise prediction of the optimal conditions that maximize the biosynthesis of AuNPs, making them effective for extremely precise prediction of future nanomaterials properties. The biosynthesized AuNPs were characterized to investigate the optical, morphological, and chemical properties. All characterization tests confirmed the suitability and efficacy of the cell-free supernatant of *Streptomyces flavolimosus* as bio-converter agent. The results demonstrated a significant inhibitory effect of biogenic AuNPs on the proliferation of both MCF-7 human breast cancer and Hela carcinoma cell lines. Further, AuNPs showed potential inhibitory effect against growth of tumor in tumor-bearing mice models. AuNPs significantly reduced the tumor volume, tumor weight, and decreased number of viable tumor cells in EAC bearing mice. This study showed that the co-treatment with the biogenic AuNPs and DOX enhance the inhibitory effect efficacy of DOX. It is recommended to study the mechanism of the co-treatment therapy-induced cytotoxicity to fully elucidate the enhanced anticancer effect of the co-treatment therapy.

### Supplementary Information

Supplementary Figures.

## Data Availability

All data generated or analyzed during this study are included in this article.

## References

[1] El-Naggar NE, Abdelwahed NA, Darwesh OM (2014). Fabrication of biogenic antimicrobial silver nanoparticles by *Streptomyces aegyptia* NEAE 102 as eco-friendly nanofactory. J. Microbiol. Biotechnol..

[2] El-Naggar NE, Saber WI, Zweil AM, Bashir SI (2022). An innovative green synthesis approach of chitosan nanoparticles and their inhibitory activity against phytopathogenic *Botrytis cinerea* on strawberry leaves. Sci. Rep..

[3] Menon S, Rajeshkumar S, Kumar V (2017). A review on biogenic synthesis of gold nanoparticles, characterization, and its applications. Resour.-Eff. Technol..

[4] Pissuwan D, Niidome T, Cortie BM (2011). The forthcoming applications of gold nanoparticles in drug and gene delivery systems. J. Control Release.

[5] Cai W, Gao T, Hong H, Sun J (2008). Applications of gold nanoparticles in cancer nanotechnology. Nanotechnol. Sci. Appl..

[6] Giasuddin A, Jhuma K, Haq A (2012). Use of gold nanoparticles in diagnostics, surgery and medicine: A review. Bangladesh J. Med. Biochem..

[7] Nune SK (2009). Green nanotechnology from tea: Phytochemicals in tea as building blocks for production of biocompatible gold nanoparticles. J. Mater. Chem..

[8] Doria G (2012). Noble metal nanoparticles for biosensing applications. Sensors.

[9] Cabuzu D, Cirja A, Puiu R, Grumezescu AM (2015). Biomedical applications of gold nanoparticles. Curr. Top. Med. Chem..

[10] Kirtana A, Abdul R, Barathi S, Afaq S, Malik A, Tarique M (2022). Nanotechnology and its applications in molecular detection. Application of Nanoparticles in Tissue Engineering.

[11] Anik MI, Mahmud N, Al Masud A, Hasan M (2022). Gold nanoparticles (GNPs) in biomedical and clinical applications: A review. Nano Select.

[12] Alomari G, Hamdan S, Al-Trad B (2021). Gold nanoparticles as a promising treatment for diabetes and its complications: Current and future potentials. Braz. J. Pharm. Sci..

[13] Mikhailova EO (2021). Gold nanoparticles: Biosynthesis and potential of biomedical application. J. Funct. Biomater..

[14] El-Naggar NE, Mohamedin A, Hamza SS, Sherief AD (2016). Extracellular biofabrication, characterization, and antimicrobial efficacy of silver nanoparticles loaded on cotton fabrics using newly isolated *Streptomyces* sp. SSHH-1E. J. Nanomater..

[15] Mohamedin A, El-Naggar NE, Shawqi Hamza S, Sherief AA (2015). Green synthesis, characterization and antimicrobial activities of silver nanoparticles by *Streptomyces viridodiastaticus* SSHH-1 as a living nanofactory: Statistical optimization of process variables. Curr. Nanosci..

[16] Barnawi N, Allehyani S, Seoudi R (2022). Biosynthesis and characterization of gold nanoparticles and its application in eliminating nickel from water. J. Mater. Res. Technol..

[17] Tikariha S, Singh S, Banerjee S, Vidyarthi AS (2012). Biosynthesis of gold nanoparticles, scope and application: A review. Int. J. Pharm. Sci. Res..

[18] Kitching M, Ramani M, Marsili E (2015). Fungal biosynthesis of gold nanoparticles: mechanism and scale up. Microb. Biotechnol..

[19] El-Naggar NE, Shiha AM, Mahrous H, Mohammed AB (2022). Green synthesis of chitosan nanoparticles, optimization, characterization and antibacterial efficacy against multi drug resistant biofilm-forming *Acinetobacter baumannii*. Sci. Rep..

[20] Golinska P (2014). Biogenic synthesis of metal nanoparticles from actinomycetes: Biomedical applications and cytotoxicity. Appl. Microbiol. Biotechnol..

[21] Mosmann T (1983). Rapid colorimetric assay for cellular growth and survival: Application to proliferation and cytotoxicity assays. J. Immunol. Methods.

[22] Elsherbiny NM, Younis NN, Shaheen MA, Elseweidy MM (2016). The synergistic effect between vanillin and doxorubicin in ehrlich ascites carcinoma solid tumor and MCF-7 human breast cancer cell line. Pathol. Res. Pract..

[23] El-Naggar NE, Soliman HM, El-Shweihy NM (2018). Extracellular cholesterol oxidase production by *Streptomyces aegyptia*, in vitro anticancer activities against rhabdomyosarcoma, breast cancer cell-lines and in vivo apoptosis. Sci. Rep..

[24] Sharawi Z (2020). WTherapeutic effect of *Arthrocnemum machrostachyum* methanolic extract on Ehrlich solid tumor in mice. BMC Complement. Med. Ther..

[25] Khan MAR (2022). A review on gold nanoparticles: Biological synthesis, characterizations, and analytical applications. Results Chem..

[26] Botteon CEA (2021). Biosynthesis and characterization of gold nanoparticles using Brazilian red propolis and evaluation of its antimicrobial and anticancer activities. Sci. Rep..

[27] Ahmad A (2003). Extracellular biosynthesis of silver nanoparticles using the fungus *Fusarium oxysporum*. Colloids Surf. B.

[28] Doan VD (2020). Biosynthesis of silver and gold nanoparticles using aqueous extract of *Codonopsis pilosula* roots for antibacterial and catalytic applications. J. Nanomater..

[29] Kalabegishvili TL (2011). Characterization of microbial synthesis of silver and gold nanoparticles with electron microscopy techniques. J. Adv. Microsc. Res..

[30] Khadivi Derakhshan F, Dehnad A, Salouti M (2012). Extracellular biosynthesis of gold nanoparticles by metal resistance bacteria: *Streptomyces griseus*. Synth. React. Inorg. Met.-Org. Nano-Met. Chem..

[31] Sobczak-Kupiec A, Malina D, Zimowska M, Wzorek Z (2011). Characterization of gold nanoparticles for various medical application. Dig. J. Nanomater. Biostruct..

[32] Gupta R (2020). Crystal growth and kinetic behaviour of *Pseudoalteromonas espejiana* assisted biosynthesized gold nanoparticles. Oxid. Med. Cell. Longev..

[33] El-Naggar NEA, Bashir SI, Rabei NH, Saber WI (2022). Innovative biosynthesis, artificial intelligence-based optimization, and characterization of chitosan nanoparticles by *Streptomyces microflavus* and their inhibitory potential against *Pectobacterium carotovorum*. Sci. Rep..

[34] Rónavári A (2021). Green silver and gold nanoparticles: Biological synthesis approaches and potentials for biomedical applications. Molecules.

[35] Huang Q (2019). Disinfection efficacy of green synthesized gold nanoparticles for medical disinfection applications. Afr. Health Sci..

[36] Cai F, Li J, Sun J, Ji Y (2011). Biosynthesis of gold nanoparticles by biosorption using *Magnetospirillum gryphiswaldense* MSR-1. Chem. Eng. J..

[37] Rajeshkumar S (2016). Anticancer activity of eco-friendly gold nanoparticles against lung and liver cancer cells. J. Genet. Eng. Biotechnol..

[38] Akhtar S (2022). Formulation of gold nanoparticles with hibiscus and curcumin extracts induced anti-cancer activity. Arab. J. Chem..

[39] Suriyakala G (2022). Green synthesis of gold nanoparticles using *Jatropha integerrima* Jacq. flower extract and their antibacterial activity. J. King Saud Univ. Sci..

[40] Gong J, Li J, Xu J, Xiang Z, Mo L (2017). Research on cellulose nanocrystals produced from cellulose sources with various polymorphs. RSC Adv..

[41] Amini R, Brar SK, Cledon M, Surampalli RY (2016). Intertechnique comparisons for nanoparticle size measurements and shape distribution. J. Hazard Toxic Radioact. Waste.

[42] Kim A, Ng WB, Bernt W, Cho NJ (2019). Validation of size estimation of nanoparticle tracking analysis on polydisperse macromolecule assembly. Sci. Rep..

[43] Muthuvel A, Adavallan K, Balamurugan K, Krishnakumar N (2014). Biosynthesis of gold nanoparticles using *Solanum nigrum* leaf extract and screening their free radical scavenging and antibacterial properties. Biomed. Prev. Nutr..

[44] Bhambure R, Bule M, Shaligram N, Kamat M, Singhal R (2009). Extracellular biosynthesis of gold nanoparticles using *Aspergillus niger—*Its characterization and stability. Chem. Eng. Technol..

[45] Owaid MN, Rabeea MA, Aziz AA, Jameel MS, Dheyab MA (2019). Mushroom-assisted synthesis of triangle gold nanoparticles using the aqueous extract of fresh *Lentinula edodes* (shiitake), Omphalotaceae. Environ. Nanotechnol. Monit. Manag..

[46] Sathiyaraj S (2021). Biosynthesis, characterization, and antibacterial activity of gold nanoparticles. J. Infect. Public Health.

[47] Honary S, Zahir F (2013). Effect of zeta potential on the properties of nano-drug delivery systems—A review (Part 2). Trop. J. Pharm. Res..

[48] Gumustas M, Sengel-Turk CT, Gumustas A, Ozkan SA, Uslu B, Grumezescu AM (2017). Effect of polymer-based nanoparticles on the assay of antimicrobial drug delivery systems. Multifunctional Systems for Combined Delivery, Biosensing and Diagnostics.

[49] Smith MC, Crist RM, Clogston JD, McNeil SE (2017). Zeta potential: A case study of cationic, anionic, and neutral liposomes. Anal. Bioanal. Chem..

[50] Amina SJ, Guo B (2020). A review on the synthesis and functionalization of gold nanoparticles as a drug delivery vehicle. Int. J. Nanomed..

[51] Pugh RJ, Matsunaga T, Fowkes FM (1983). The dispersibility and stability of carbon black in media of low dielectric constant. 1. Electrostatic and steric contributions to colloidal stability. Colloids Surf..

[52] Tsalsabila A, Herbani Y, Sari YW (2022). Study of lysine and asparagine as capping agent for gold nanoparticles. J. Phys. Conf. Ser..

[53] Gopinath K, Gowri S, Karthika V, Arumugam A (2014). Green synthesis of gold nanoparticles from fruit extract of *Terminalia arjuna*, for the enhanced seed germination activity of *Gloriosa superba*. J. Nanostruct. Chem..

[54] Palanivel, V., Jaehong, S., KeukSoo, B. & Byung-Taek, O. *Gold Nanoparticles Mediated Coloring of Fabrics and Leather for Antibacterial Activity* (2016). 10.1016/j.jphotobiol.2016.03.051 27104665

[55] Manivasagan P, Oh J (2015). Production of a novel fucoidanase for the green synthesis of gold nanoparticles by *Streptomyces* sp. and its cytotoxic effect on HeLa cells. Mar. Drugs.

[56] Marimuthu S (2011). Evaluation of green synthesized silver nanoparticles against parasites. Parasitol. Res..

[57] Shaik MR (2017). Green synthesis and characterization of palladium nanoparticles using *Origanum vulgare* L. extract and their catalytic activity. Molecules.

[58] El-Nour KMA, Salam ETA, Soliman HM, Orabi A (2017). Gold nanoparticles as a direct and rapid sensor for sensitive analytical detection of biogenic amines. Nanoscale Res. Lett.

[59] Chaber R (2021). A preliminary study of FTIR spectroscopy as a potential non-invasive screening tool for pediatric precursor B lymphoblastic leukemia. Molecules.

[60] Srinath BS, Rai VR (2015). Rapid biosynthesis of gold nanoparticles by *Staphylococcus epidermidis*: Its characterization and catalytic activity. Mater. Lett..

[61] Tajammul Hussain S, Iqbal M, Mazhar M (2009). Size control synthesis of starch capped gold nanoparticles. J. Nanopart. Res..

[62] Correa-Llantén DN, Muñoz-Ibacache SA, Castro ME, Muñoz PA, Blamey JM (2013). Gold nanoparticles synthesized by *Geobacillus* sp. strain ID17 a thermophilic bacterium isolated from Deception Island. Antarctica. Microb. Cell Factories.

[63] Divakaran D (2019). fruit extract capped gold nanoparticles: Synthesis and their differential cytotoxicity effect on breast cancer cells. Mater. Lett..

[64] Priya Velammal S, Devi TA, Amaladhas TP (2016). Antioxidant, antimicrobial and cytotoxic activities of silver and gold nanoparticles synthesized using *Plumbago zeylanica* bark. J. Nano Chem..

[65] Ristig S, Kozlova D, Meyer-Zaika W, Epple M (2014). An easy synthesis of autofluorescent alloyed silver–gold nanoparticles. J. Mater. Chem. B.

[66] Składanowski M (2017). Silver and gold nanoparticles synthesized from *Streptomyces* sp. isolated from acid forest soil with special reference to its antibacterial activity against pathogens. J. Clust. Sci..

[67] Ranjitha VR, Rai VR (2017). Actinomycetes mediated synthesis of gold nanoparticles from the culture supernatant of *Streptomyces griseoruber* with special reference to catalytic activity. 3 Biotech.

[68] El-Naggar NE (2015). Isolation, screening and identification of actinobacteria with uricase activity: Statistical optimization of fermentation conditions for improved production of uricase by *Streptomyces rochei* NEAE–25. Int. J. Pharmacol..

[69] El-Naggar NE, Hamouda RA, El-Khateeb AY, Rabei NH (2021). Simultaneous bioremediation of cationic copper ions and anionic methyl orange azo dye by brown marine alga *Fucus vesiculosus*. Sci. Rep..

[70] El-Naggar NE, El-Khateeb AY, Ghoniem AA, El-Hersh MS, Saber WI (2020). Innovative low-cost biosorption process of Cr^6+^ by *Pseudomonas alcaliphila* NEWG-2. Sci. Rep..

[71] El-Naggar NE, Moawad H, Abdelwahed NA (2017). Optimization of fermentation conditions for enhancing extracellular production of L-asparaginase, an anti-leukemic agent, by newly isolated *Streptomyces brollosae* NEAE-115 using solid state fermentation. Ann. Microbiol..

[72] El-Naggar NE, Haroun SA, El-Weshy EM, Metwally EA, Sherief AA (2019). Mathematical modeling for bioprocess optimization of a protein drug, uricase, production by *Aspergillus welwitschiae* strain 1–4. Sci. Rep..

[73] Kulkarni N, Muddapur U (2014). Biosynthesis of metal nanoparticles: A review. J. Nanotechnol..

[74] Camas M, Celik F, Sazak Camas A, Ozalp HB (2019). Biosynthesis of gold nanoparticles using marine bacteria and Box–Behnken design optimization. Part. Sci. Technol..

[75] Zonooz NF, Salouti M, Shapouri R, Nasseryan J (2012). Biosynthesis of gold nanoparticles by *Streptomyces* sp. ERI-3 supernatant and process optimization for enhanced production. J. Clust. Sci..

[76] Saha N, Gupta SD (2016). Biogenic synthesis and structural characterization of polyshaped gold nanoparticles using leaf extract of *Swertia chirata* along with process optimization by response surface methodology (RSM). J. Clust. Sci..

[77] Shivaji SW, Arvind MD, Zygmunt S (2014). Biosynthesis, optimization, purification and characterization of gold nanoparticles. Afr. J. Microbiol. Res..

[78] Shakouri V, Salouti M, Mohammadi B, Zonooz NF (2016). Procedure optimization for increasing biosynthesis rate of gold nanoparticles by Aspergillus flavus Supernatant. Synth. React. Inorg. Met.-Org. Nano-Met. Chem..

[79] Al-Khattaf FS (2021). Gold and silver nanoparticles: Green synthesis, microbes, mechanism, factors, plant disease management and environmental risks. Saudi J. Biol. Sci..

[80] Priyadarshini E, Pradhan N, Sukla LB, Panda PK (2014). Controlled synthesis of gold nanoparticles using *Aspergillus terreus* IF0 and its antibacterial potential against Gram negative pathogenic bacteria. J. Nanotechnol..

[81] Virmani I (2020). Comparative anticancer potential of biologically and chemically synthesized gold nanoparticles. J. Clust. Sci..

[82] Munawer U (2020). Biofabrication of gold nanoparticles mediated by the endophytic Cladosporium species: Photodegradation, in vitro anticancer activity and in vivo antitumor studies. Int. J. Pharm..

[83] Datkhile KD, Durgavale PP, Patil MN, Jagdale NJ, Deshmukh VN (2021). Biosynthesis, characterization and evaluation of biological properties of biogenic gold nanoparticles synthesized using *Nothapodytes foetida* leaf extract. Nanosci. Nanotechnol. Asia.

[84] Raghavan BS, Kondath S, Anantanarayanan R, Rajaram R (2015). Kaempferol mediated synthesis of gold nanoparticles and their cytotoxic effects on MCF-7 cancer cell line. Process. Biochem..

[85] Kalaivani R (2020). Chitosan mediated gold nanoparticles against pathogenic bacteria, fungal strains and MCF-7 cancer cells. Int. J. Biol. Macromol..

[86] Clarance P (2020). Green synthesis and characterization of gold nanoparticles using endophytic fungi *Fusarium solani* and its in-vitro anticancer and biomedical applications. Saudi J. Biol. Sci..

[87] Asl SS, Tafvizi F, Noorbazargan H (2022). Biogenic synthesis of gold nanoparticles using *Satureja rechingeri* Jamzad: A potential anticancer agent against cisplatin-resistant A2780CP ovarian cancer cells. Environ. Sci. Pollut. Res..

[88] Abdulateef SA (2023). Rapid synthesis of bovine serum albumin-conjugated gold nanoparticles using pulsed laser ablation and their anticancer activity on Hela cells. Arab. J. Chem..

[89] Pan Y (2009). Gold nanoparticles of diameter 1.4 nm trigger necrosis by oxidative stress and mitochondrial damage. Small.

[90] Dickson J, Weaver B, Vivekanand P, Basu S (2023). Anti-neoplastic effects of gold nanoparticles Synthesized using green sources on cervical and melanoma cancer cell lines. Bio Nano Sci..

[91] Aboyewa JA, Sibuyi NR, Goboza M, Murtz LA, Oguntibeju OO, Meyer M (2022). Co-treatment of Caco-2 cells with doxorubicin and gold nanoparticles produced from *Cyclopia intermedia* extracts or mangiferin enhances drug effects. Nanomaterials.

[92] Lim ZZJ, Li JEJ, Ng CT, Yung LYL, Bay BH (2011). Gold nanoparticles in cancer therapy. Acta Pharmacol. Sin..

[93] Lee J, Chatterjee DK, Lee MH, Krishnan S (2014). Gold nanoparticles in breast cancer treatment: Promise and potential pitfalls. Cancer Lett..

[94] Yahyaei B, Nouri M, Bakherad S, Hassani M, Pourali P (2019). Effects of biologically produced gold nanoparticles: Toxicity assessment in different rat organs after intraperitoneal injection. AMB Express.

[95] Ibrahim B, Akere TH, Chakraborty S, Valsami-Jones E, Ali-Boucetta H (2023). Gold nanoparticles induced size dependent cytotoxicity on human alveolar adenocarcinoma cells by inhibiting the ubiquitin proteasome system. Pharmaceutics.

[96] Zhang XD (2010). Toxicologic effects of gold nanoparticles in vivo by different administration routes. Int. J. Nanomed..

[97] Shukla R, Bansal V, Chaudhary M, Basu A, Bhonde RR, Sastry M (2005). Biocompatibility of gold nanoparticles and their endocytotic fate inside the cellular compartment: A microscopic overview. Langmuir.

[98] Yah CS (2013). The toxicity of gold nanoparticles in relation to their physiochemical properties. Biomed. Res..

[99] Semmler-Behnke M (2008). Biodistribution of 1.4- and 18-nm gold particles in rats. Small.

[100] De Jong WH (2008). Particle size-dependent organ distribution of gold nanoparticles after intravenous administration. Biomaterials.

[101] Abdelhalim MAK, Moussa SAA (2013). The gold nanoparticle size and exposure duration effect on the liver and kidney function of rats: In vivo. Saudi J. Biol. Sci..

[102] Zhu GH, Gray AB, Patra HK (2022). Nanomedicine: Controlling nanoparticle clearance for translational success. Trends Pharmacol. Sci..

[103] Chithrani BD, Chan WC (2007). Elucidating the mechanism of cellular uptake and removal of protein-coated gold nanoparticles of different sizes and shapes. Nano Lett..

[104] Hainfeld JF, Smilowitz HM, O’connor MJ, Dilmanian FA, Slatkin DN (2013). Gold nanoparticle imaging and radiotherapy of brain tumors in mice. Nanomedicine.

