# Supplementary material for: Artificial neural network approach for prediction of AuNPs biosynthesis by Streptomyces flavolimosus, characterization, antitumor potency in-vitro and in-vivo against Ehrlich ascites carcinoma
Source: Sci Rep. 2023 Aug 4;13:12686. doi: 10.1038/s41598-023-39177-4 (PMC10403537; doi:10.1038/s41598-023-39177-4)
Supplement: Supplementary file 1 — Supplementary Figures. [file 41598_2023_39177_MOESM1_ESM.pdf]

Artificial neural network approach for prediction of AuNPs biosynthesis by *Streptomyces flavolimosus*, characterization, antitumor potency *in-vitro* and *in-vivo* against EAC tumor mass

[1]Noura El-Ahmady El-Naggar, [1]Nashwa H. Rabei, Mohamed F. Elmansy[2], Omar T. Elmessiry[2], Mostafa K. El-Sherbeny[2], Mohanad E. El-Saidy[2], Mohamed T. Sarhan[2], Manar G. Helal[3]

[1]Department of Bioprocess Development, Genetic Engineering and Biotechnology Research Institute, City of Scientific Research and Technological Applications (SRTA-City), Alexandria 21934, Egypt.
[2]Biotechnology and Its Application Program, Department of Botany, Faculty of Science, Mansoura University, Mansoura 35516, Egypt.
[3]Department of Pharmacology and Toxicology, Faculty of Pharmacy, Mansoura University, Mansoura 35516, Egypt.

Corresponding Author's information

Dr. Noura El-Ahmady Ali El-Naggar

Address:

Bioprocess Development Department,

Genetic Engineering and Biotechnology Research Institute,

City of Scientific Research and Technological Applications,

New Borg El- Arab City, 21934, Alexandria, Egypt

Tel: (002)01003738444

Fax: (002)03 4593423

E-mail: nouraalahmady@yahoo.com

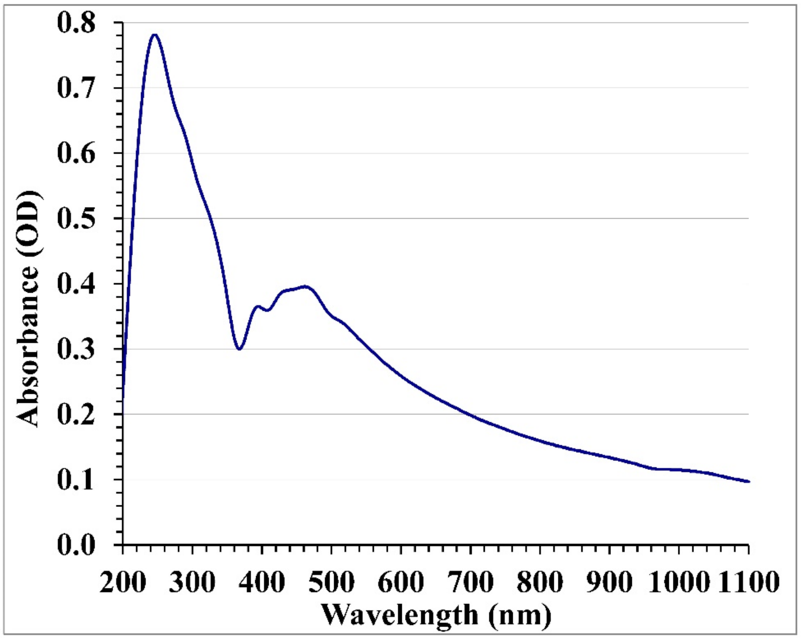

Supplementary Figure S 1. The absorbance spectrum of the cell-free supernatant.

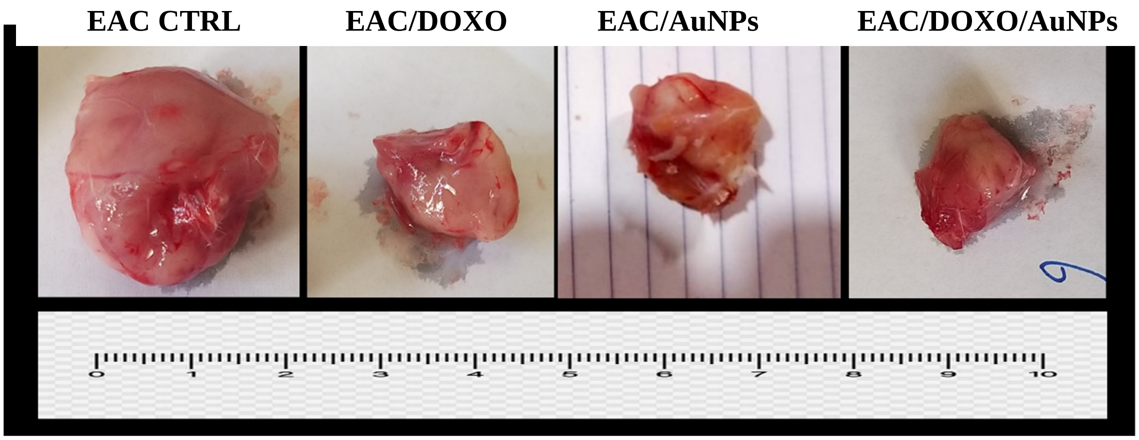

Supplementary Figure S 2. raw tumor images, all tumor images (**Figure 10** C) were taken at the same magnification power, zooming and distance from camera.
